# Long‐Term Outcome of Complex Regional Pain Syndrome versus Limb Pain of Other Origin: Results From a Telephone Survey With up to 5‐year Follow‐Up

**DOI:** 10.1155/prm/4722836

**Published:** 2026-01-21

**Authors:** Kübra Arslan, Andrea Westermann, Andreas Schwarzer, Christoph Maier, Lionel Butry, Johannes Forsting, Elena Enax-Krumova

**Affiliations:** ^1^ Department of Pain Medicine, BG University Hospital Bergmannsheil gGmbH, Ruhr University Bochum, Bochum, Germany, ruhr-uni-bochum.de; ^2^ University Hospital of Pediatrics and Adoslescent Medicine, St. Josef Hospital Bochum, Ruhr University Bochum, Bochum, Germany, ruhr-uni-bochum.de; ^3^ Department of Neurology, BG University Hospital Bergmannsheil gGmbH, Ruhr University Bochum, Bochum, Germany, ruhr-uni-bochum.de

**Keywords:** complex regional pain syndrome, nociceptive limb pain, pain disability, posttraumatic neuralgia, real-world data, treatment outcome, work-related disability

## Abstract

**Background:**

Complex regional pain syndrome (CRPS) is a disabling condition requiring long‐term treatment. This study evaluated long‐term outcomes in CRPS patients compared to posttraumatic neuralgia (PN) and nociceptive limb pain (NLP) 1–5 years after diagnosis and treatment initiation in a tertiary clinic.

**Methods:**

Patients with CRPS lasting more than 1 year (*n* = 116), PN (*n* = 68), or NLP (*n* = 75) participated in a standardized telephone interview assessing pain intensity, functional impairment, neglect‐like symptoms, and disability in daily life and work.

**Results:**

PN patients had higher pain intensity compared to CRPS (NRS 5.7 ± 2.1 vs. 4.5 ± 2.6; main effect: *p* = 0.005; post hoc test: *p* = 0.003). Functional outcomes varied significantly between groups (*p* < 0.001). CRPS patients showed the highest rate of severe impairment (71%), especially in those with ≥ 3 clinical signs, while outcome was better in NLP (*p* ≤ 0.027), especially in part‐time workers. The global impression of disability varied between groups (*p* = 0.044): PN patients reported a high level of severe disability (70%), while CRPS had the most subjects with “none/slight” disability (23%). Pain and function were tightly linked to perceived disability in all groups. Neither the neglect‐like total score (CRPS: 2.4 (2.2–2.6); neuralgia: 2.5 (2.3–2.7); NLP: 2.2 (1.9–2.5); *p* = 0.055) nor the working ability differed between groups (*p* = 0.397); however, only in CRPS, neglect‐like symptoms were associated with higher pain intensity and global disability, as well as lower functional outcome. CRPS patients treated with corticosteroids (*n* = 55) had more positive clinical signs at baseline and poorer outcomes than patients without corticosteroids (*n* = 61).

**Conclusions:**

Long‐term impairment, work‐related, and global disability in CRPS were similar to those of neuralgia and NLP patients treated in a tertiary pain clinic. Despite shorter disease duration, CRPS patients more often report severe disability, while pain is comparable to other chronic limb pain courses. More pronounced positive signs in CRPS at baseline are associated with poor outcomes. These findings emphasize the heterogeneous course of CRPS and the importance of individualized multidimensional approaches.

## 1. Introduction

Complex regional pain syndrome (CRPS) is a rare but severe complication after trauma, such as distal radius fractures [1]. It is characterized by severe pain disproportionate to the initiating event. CRPS is being clinically diagnosed according to the Budapest criteria, including signs and symptoms of edema, sensory, motor and autonomic dysfunctions, and impaired joint function with varying severity [2]. Its exact pathophysiology is still not completely understood. The prognosis exhibits high variability and is influenced by various factors, including the type and start of treatment, the mental state of the patients, and the predominant symptoms [3–5]. Nevertheless, data on the long‐term implications in everyday life, particularly with high direct healthcare costs and working incapacity, are rare [6].

An early diagnosis and treatment start is favorable for the prognosis of CRPS. Especially, within the first 6 months, a significant improvement in pain, function, and ability to work can be achieved. In contrast, after this time, the improvement is often limited [7]. However, recent data suggest that rehabilitation benefits CRPS patients, regardless of symptom duration [8]. Nevertheless, a substantial proportion of CRPS patients develop long‐lasting symptoms, including both chronic pain and functional disability, leading to psychosocial problems [4, 7]. Work‐related disability appears to persist for up to 5 years in one‐third of patients following disease onset [9]. Baseline anxiety, pain intensity, and functional status could predict CRPS severity in the upcoming years in a cohort with an 8‐year follow‐up [10].

While the long‐term prognosis of CRPS regarding life quality remains limited, comparative data, especially against other patient groups with similar complaints, are scarce [11]. There is a lack of long‐term data on return to work, daily functioning, social impact, and overall quality of life in CRPS, particularly compared to patients suffering from other forms of posttraumatic or limb‐related chronic pain.

Therefore, our study aimed to investigate the long‐term outcomes of CRPS patients compared to chronic pain patients with limb pain of other origins, such as neuropathic pain or nociceptive limb pain (NLP), focusing on pain intensity, functional impairment, and ability to work. These data could contribute to a better understanding of the treatment effects and the long‐lasting complications of the daily life of the studied entities.

## 2. Methods

### 2.1. Study Design and Patients’ Inclusion and Exclusion Criteria

Following approval by the local Ethics Committee of the Medical Faculty, Ruhr University Bochum, Germany (Ref. 17‐6202), a database analysis of the tertiary pain clinic at the Department of Pain Medicine, BG University Hospital Bergmannsheil, identified 604 eligible patients who were treated between 1 and 5 years prior as potential candidates for a telephone interview from 2014 to 2016.

**Figure 1:**
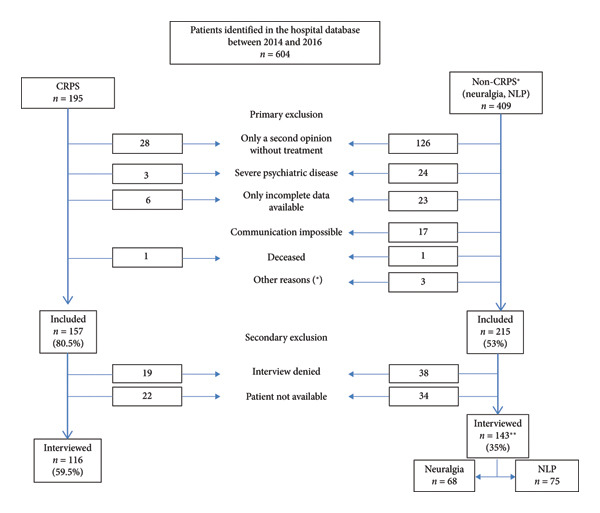
STROBE diagram of the study procedure. CRPS = complex regional pain syndrome; NLP = nociceptive limb pain.

The clinic’s pain specialists independently performed all diagnoses and treatments and were not part of the study. For all CRPS patients, a Technetium‐99 m 3‐phase scintigraphy was available [12]. The inclusion criteria were as follows: at least one documented clinical and radiological examination between 2014 and 2016, pain treatment in the department for at least 1 year, age over 18 years, and informed consent. Primary and secondary exclusion criteria are detailed in Figure 1. A total of 372 patients fulfilled the inclusion criteria and received a written invitation for the telephone interview and a centimeter scale (see Figure 1). All patients provided consent and completed a 25‐min structured interview, which included validated questionnaires and custom‐developed questions (see Supporting file 1).

Data were collected at three points: initial presentation at the pain clinic (T0), last clinic visit (T1), and telephone interview (T2). Clinical data were extracted from hospital databases (QUAST, Medico) and converted into scores for further analysis.

The three patient groups were further divided into subgroups based on the affected extremity (upper/lower extremities) to enhance comparability.

### 2.2. Clinical Findings (T0, T1)

Clinical findings were analyzed separately for the upper and lower limbs.

1. Sensory impairment: abnormal sensation, results of the nerve conduction studies, or quantitative sensory testing (QST).
2. Vasomotor/trophic changes: excessive sweating of the affected limb or edema.
3. Functional impairment: incomplete fist closure in the upper limb, limited force of plantarflexion/dorsiextension, and ankle mobility in the lower limb.

### 2.3. Pain and Pain Treatment (T0, T1, T2)

Average pain intensity was assessed using the 11‐point Numerical Rating Scale (NRS; 0 = no pain, 10 = worst imaginable pain). Based on the NRS, pain intensity was categorized as none (0), low (1–3), moderate (4–6), and severe (≥ 7).

Aspects of multimodal pain management were systematically assessed at all three time points (T0, T1, and T2). This included pharmacological treatment such as nonsteroidal anti‐inflammatory drugs (NSAIDs), metamizole, tricyclic antidepressants, selective serotonin reuptake inhibitors (SSRIs), selective serotonin–norepinephrine reuptake inhibitors (SSNRIs), opioids, anticonvulsants, and topically applied agents. Additional therapies comprised prednisolone use (T1), physiotherapy, and occupational therapy (T1, T2). The use of orthoses and other medical aids was recorded throughout the observation period.

Interventional procedures, documented at T1 and T2, included nerve and joint injections, plexus or spinal catheterization, sympathetic nerve blocks (e.g., stellate ganglion blocks), and neuromodulation techniques such as spinal cord or peripheral nerve stimulation. Alternative treatments such as acupuncture, shockwave therapy, and lymphatic drainage were recorded at T2. Moreover, external treatments outside the Department of Pain Medicine were documented at T2, including additional injections, surgical interventions, and neurostimulation procedures. Patients were also specifically asked about any surgeries performed on the affected limb during the observation period and their perceived outcomes.

Furthermore, the frequency of medication use was assessed with the reported pain intensity and categorized as low or no pain (NRS ≤ 3) with or without medication, moderate pain (NRS 4–6) with or without medication, and severe pain (NRS ≥ 7), regardless of medication use.

### 2.4. Questionnaires During the Telephone Interview (T2)

All patients filled out a questionnaire, including 13 questions addressing the following aspects: pain, current medication and treatment, functionality, severity of neglect‐like symptoms (NLSs), need for aids, working ability, and external treatments. Patients also rated their global impression of impairment in daily activities, categorized as “none,” “light,” “moderate,” or “severe.”

An overall classification of functional impairment for both upper and lower limbs was derived from a structured combination of patient‐reported questionnaire data, objective functional assessments, and previously established criteria described in the literature [13–16].

#### 2.4.1. Functionality (T2)

##### 2.4.1.1. Upper Limb

Patients were asked about assistive devices (e.g., orthoses, wrist cuff, adaptive cutlery). Functional assessments included finger‐palm distance (0 cm = none; 0.1–1 cm = mild; 1.1–2.5 cm = moderate; ≥ 3 cm = severe) and thumb opposition (to digit V = none; to digits III, IV = moderate; to digit II = severe). Furthermore, the following fine motor tasks were included: stroke over the back of the head, use cutlery, lift a full coffee cup and put it down again, and turn on a bottle cap. Answers included “possible,” “reduced,” and “impossible,” and the possible causes were obtained (pain, strength, mobility, lack of movement control). The fine motor assessments were combined into a single categorical score, which was defined as follows: “none” indicated no impairment, with at most one task showing any limitation; “moderate” reflected mild to moderate impairment in at least two tasks; and “severe” denoted a complete impairment in at least one task.

In addition to the individual assessments, the severity levels of each fine motor subcategory—finger‐hollow distance, thumb opposition, and task‐specific fine motor performance—were consolidated into an overall functional severity score. This final score was defined as follows:

- None: No impairment, characterized by a finger‐hollow distance of 0 cm
- Moderate: All intermediate values that did not meet the criteria for severe impairment
- Severe: Markedly limited finger‐hollow distance (> 3 cm) or a “severe” rating in the questionnaire‐based evaluation of fine motor tasks, with the finger‐hollow distance not equal to zero.

##### 2.4.1.2. Lower Limb

Functional impairment of the lower limb was determined by stepping with the affected foot (“impossible,” “restricted,” “possible”), standing (“impossible,” “possible for a few seconds,” “possible for > 30 s”), the painless walking distance (“not possible,” “only a few steps,” “> 100 m,” “> 1 km”), and the need of aids (“none”; use of orthosis, special shoes—“moderate”; use of wheelchair, rollator or crutches—“severe”). Causes for restrictions were recorded (pain, strength, mobility, lack of movement control).

The impairment in standing was evaluated based on the patient’s ability to bear weight on the affected foot and perform specific functional tasks. It was categorized as follows:

- None: The patient could step normally on the affected foot and maintain a standing position on the heel and tiptoe for more than 30 s without using assistive devices.
- Moderate: Functional limitations that did not meet the “none” or “severe” criteria were classified as moderate.
- Severe: The patient could not bear weight on the affected foot and stand on the heel or tiptoe, regardless of whether assistive devices were used.

This assessment was combined with additional functional domains, including walking ability and the use of mobility aids, to calculate a global impairment score for the lower limb. The global score was defined as follows:

- None: No functional limitations were present across any domain.
- Moderate: At least one domain showed a moderate degree of impairment.
- Severe: At least one domain showed severe impairment, such as the need for major mobility aids or inability to perform basic standing or walking tasks.

#### 2.4.2. Severity of NLSs

Assessment was adapted from Frettlöh et al. and Galer and Jensen, using a 5‐item Likert scale [17, 18]. Items were dichotomized: 1 = “not true,” scores ≥ 2 = “true.” Based on agreement with the five dichotomous NLS items, a final functional score was derived by combining the global impairment rating with the severity of NLSs as a composite outcome measure. To account for the added impact of NLSs, each patient’s functional impairment category was adjusted by one severity level: “none” was reclassified as “moderate,” “moderate” as “severe,” while “severe” remained unchanged.

#### 2.4.3. Ability to Work (T0, T2)

Patients employed before disease onset (T0) reported their return‐to‐work status (full/part‐time, different role) and reduced work capacity due to illness at T2.

Furthermore, reasons for nonemployment were recorded in a structured manner at T2, using the following subcategories: previously also not worked, old‐age pension, early retirement due to sickness/for other reasons, written incapacity to work due to sickness/for other reasons, unemployed without a sick note, and without work for other reasons. Patients who did not work at T0 because of retirement, school/university, or other reasons were excluded.

Additionally, a grading score of working ability was calculated based on the proportion of time the patient was able to work following disease onset. Working capacity of less than 40% of the period was classified as severely impaired, 40%–75% as moderately impaired, and > 75% as good.

### 2.5. Statistical Analysis

Descriptive statistics were used to describe each group’s characteristics. For continuous variables, the mean with the standard deviation and the median with interquartile ranges were reported. For categorical variables, the frequencies and percentages were calculated.

To compare the study groups’ clinical characteristics at baseline and outcome measures at follow‐up, an ANOVA with Bonferroni‐corrected post hoc *t*‐tests was performed for continuous variables. For categorical variables, the individual clinical characteristics and outcome measures between the groups were compared using the chi‐squared test or Fisher’s exact test when the assumptions for the chi‐squared test were not met.

Furthermore, each group’s response to the specialized pain therapy was examined by comparing the mean pain intensity ratings at all three time points using a repeated‐measures ANOVA with Bonferroni‐corrected post hoc *t*‐tests. NLSs were assessed using an ANCOVA with age, gender, pain duration, and pain intensity as covariates [17]. Furthermore, the binary data from the dichotomous approach were used to compare the frequency distribution between the CRPS and control groups.

To identify predictors for a good outcome in all study groups, defined by none, slight, or moderate global impression of disability, a multivariate logistic regression analysis was performed to identify significant independent risk factors. Differences between CRPS patients with and without corticosteroid treatment were examined. Additionally, changes over time in the frequency distribution of functional status (based on finger‐palm distance) and pain intensity were analyzed using the Stuart–Maxwell test.

To examine the interrelationships among key clinical variables, we performed an exploratory network analysis, constructing separate network models for each diagnostic group. For each group, five target variables were selected based on clinical relevance: Neglect Total Score, functional outcome, pain intensity at T2, working ability, and global impression of disability. Variables were converted to a numeric format to ensure compatibility across modeling steps.

All statistical analyses were performed using R (Version 4.5.0, R Foundation for Statistical Computing, Vienna, Austria). Networks were estimated using the partial correlation approach (qgraph package, Version 1.9.8, R) which models conditional dependencies between variables by statistically controlling for all others in the network.

A *p*‐value less than 0.05 was considered statistically significant.

## 3. Results

### 3.1. Patients and Demographic and Clinical Data at T0

604 patients were initially identified as potential candidates for the telephone interview (CRPS: *n* = 195; controls *n* = 409). Of these, 375 patients (CRPS: *n* = 157; controls: *n* = 215) met the primary inclusion criteria for the interview. After excluding patients who declined participation or were unavailable, 262 ultimately participated in a telephone interview (Figure 1).

All 116 patients from the CRPS group fulfilled the Budapest diagnostic criteria and presented with typical scintigraphic signs (63 female; mean age 54 years; 84% upper limb involvement). No significant differences in age, gender, BMI, or occupation before disease were found between study groups (*p* > 0.05; see Table 1). While the affected side and dominant side were comparable in both groups, the upper extremity was significantly more often affected in CPRS (84%) compared to neuralgia (62%) and NLP (32%; *p* ≤ 0.006) and in neuralgia compared to NLP (*p* = 0.003). The most common initiating factor in CPRS (90%) and neuralgia (59%) was acute trauma, while posttraumatic alterations were most common in NLP (41%). The disease duration of CRPS patients was significantly lower than that of the control group (CRPS: 0.6 ± 0.5; neuralgia: 5.7 ± 8; NLP: 3.5 ± 4.9 years; *p* < 0.001). Mean pain intensity at referral was significantly lower in the CRPS group compared to NLP (CRPS: 5.41 ± 2.05; NLP: 6.26 ± 1.79; *p* = 0.012), while pain intensity in neuralgia was comparable to CRPS and NLP (6.04 ± 1.73; *p* ≥ 0.111). CRPS patients took less medication at T0 than the control groups (*p* ≤ 0.033). A detailed overview of pharmacotherapy at all timepoints is given in Table S1. Sensory signs were significantly more frequent in CRPS (86%) and neuralgia (94%) compared to NLP (67%; *p* ≤ 0.009), while in CRPS vasomotor (72%) and sudomotor signs (83%) were more frequent. 57% of CRPS patients showed four positive clinical signs compared to 14% in neuralgia and 12% in NLP (*p* ≤ 0.001).

**Table 1:** Clinical data at time of referral (T0).

Variable	*N*	CRPS *N* = 116	Neuralgia *N* = 68	NLP *N* = 75	*p*‐value
Age	259				0.3
Mean ± SD		54 ± 12	52 ± 11	52 ± 14	
Median (Q1–Q3)		55 (48–63)	53 (48–59)	53 (41–60)	
Gender	259				0.059
Female		63 (54%)	38 (56%)	29 (39%)	
Male		53 (46%)	30 (44%)	46 (61%)	
BMI	232				0.10
Mean ± SD		26.8 ± 5.1	28.0 ± 7.2	28.8 ± 5.6	
Median (Q1–Q3)		26.1 (23.4–29.4)	26.8 (23.0–31.6)	26.8 (25.3–33.7)	
Occupation before disease	259				> 0.9
Full time		68 (59%)	42 (62%)	47 (63%)	
Part time		25 (22%)	16 (24%)	15 (20%)	
None		23 (20%)	10 (15%)	13 (17%)	
Affected extremity	259				< 0.001^#+Δ^
Lower		19 (16%)	26 (38%)	51 (68%)	
Upper		97 (84%)	42 (62%)	24 (32%)	
Affected side	259				0.3
Left		59 (51%)	33 (49%)	34 (45%)	
Right		57 (49%)	34 (50%)	38 (51%)	
Both		0 (0%)	1 (1.5%)	3 (4.0%)	
Dominant side	259				0.538
Left		9 (7.8%)	3 (4.4%)	7 (9.3%)	
Right		107 (92%)	65 (96%)	68 (91%)	
Initiating factor	259				< 0.001^#+Δ^
Acute trauma		104 (90%)	40 (59%)	28 (37%)	
Posttraumatic alteration		3 (2.6%)	15 (22%)	31 (41%)	
Other diseases		9 (7.8%)	13 (19%)	16 (21%)	
Disease duration	259				< 0.001^#+^
< 90 d		21 (18%)	5 (7.4%)	2 (2.7%)	
90–179 d		53 (46%)	7 (10%)	4 (5.3%)	
180–365 d		25 (22%)	10 (15%)	15 (20%)	
1–3 y		16 (14%)	25 (37%)	18 (24%)	
> 3 y		1 (0.9%)	21 (31%)	36 (48%)	
Mean pain intensity at T0	259				0.007^#+^
Mean ± SD		5.41 ± 2.05	6.04 ± 1.73	6.26 ± 1.79	
Median (Q1–Q3)		5 (4–7)	6 (5–7)	6 (5–8)	
Medication at T0	256				0.004^#+^
No drug		22 (19%)	9 (13%)	9 (12%)	
1 or 2 drugs		75 (66%)	33 (49%)	42 (56%)	
3 or more drugs		16 (14%)	26 (38%)	24 (32%)	
Sensory signs	256	100 (86%)	64 (94%)	48 (67%)	< 0.001^+Δ^
Vasomotor signs	242	84 (72%)	20 (32%)	20 (32%)	< 0.001^#+^
Sudomotor signs	245	96 (83%)	18 (28%)	18 (28%)	< 0.001^#+^
Motor/trophic signs	259	116 (100%)	67 (99%)	74 (99%)	0.4
Positive clinical signs	247				< 0.001^#+^
1		0 (0%)	5 (7.6%)	15 (23%)	
2		18 (16%)	32 (48%)	29 (45%)	
3		32 (28%)	20 (30%)	13 (20%)	
4		66 (57%)	9 (14%)	8 (12%)	

*Note:* *p*‐values are calculated using Pearson’s *χ* ^2^‐test or Fisher’s exact test for categorical variables, and an ANOVA with Bonferroni‐corrected *t*‐tests for continuous variables. Significant post hoc tests between groups are displayed (CRPS—Neuralgia^#^; CRPS—NLP^+^; Neuralgia—NLP^Δ^).

### 3.2. Pain and Pain Treatment Over Time

The follow‐up periods from initial presentation to the interview were similar between the groups (CRPS: 29 ± 10; neuralgia: 33 ± 11; NLP: 31 ± 13 months) but were statistically significant between CPRS and neuralgia (main effect: *p* = 0.027; post hoc test: *p* = 0.012).

**Figure 2:**
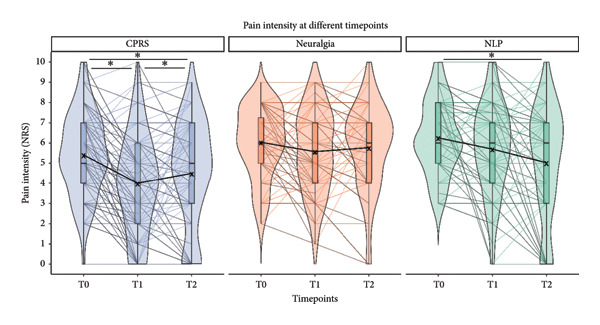
Pain intensity over time in study groups. ^∗^ *p* < 0.05 in Bonferroni‐corrected post hoc analysis after significant repeated‐measures ANOVA.

**Figure 3:**
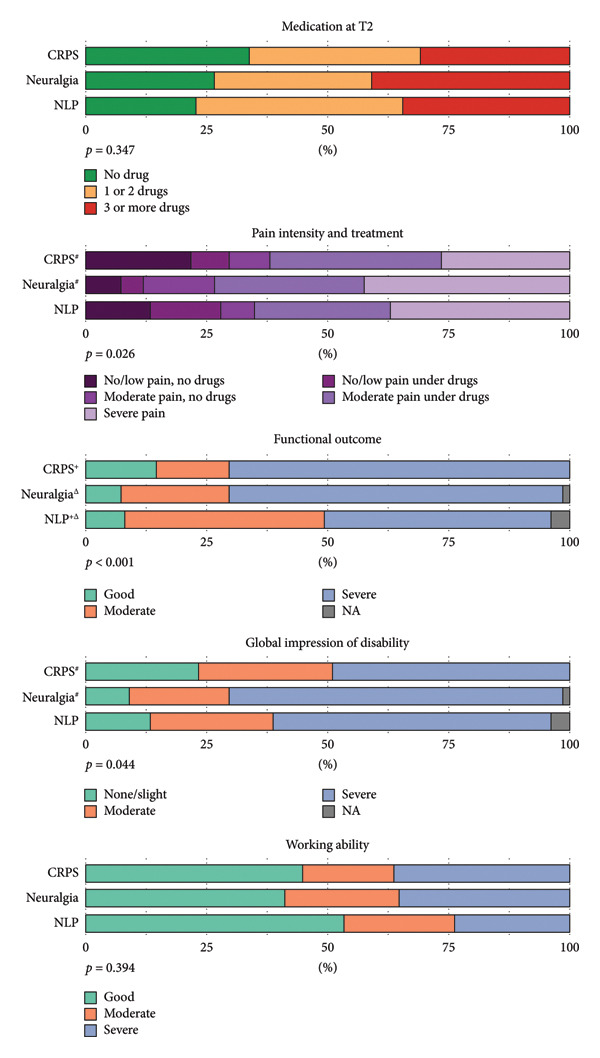
Overview of main outcome measures at follow‐up (T2). *p*‐values are calculated using Pearson’s *χ* ^2^‐test. Significant post hoc tests between groups are displayed (CRPS—Neuralgia^#^; CRPS—NLP^+^; Neuralgia—NLP^Δ^).

In the CRPS group, mean pain intensity significantly decreased over time (T0: 5.41 ± 2.05; T1: 4.00 ± 2.39; main effect: *p* < 0.001; post hoc test: *p* < 0.001), followed by a slight but significant increase at follow‐up (T2: 4.50 ± 2.60; *p* = 0.032). The neuralgia patients had a stable pain intensity over time (T0: 6.04 ± 1.73; T1: 5.57 ± 2.03; T2: 5.77 ± 2.13; *p* = 0.180). In the NLP group, a significant decrease over time was observed with significant post hoc tests between T0 and T2 (T0: 6.26 ± 1.79; T1: 5.70 ± 2.24; T2: 5.03 ± 2.77; main effect: *p* = 0.001; post hoc test: *p* = 0.001). Mean pain intensities over time are illustrated in Figure 2. At T2, patients with neuralgia had higher pain intensity than CRPS (main effect: *p* = 0.005; post hoc test: *p* = 0.003). While at T0, CPRS patients took less medication than the control groups, at T2, pharmacotherapy was comparable between the study groups (*p* = 0.347; see Table 2). When pain intensity and treatment were considered together, 22% of CRPS patients reported low pain levels without medication (neuralgia: 7.4%; NLP: 13%), while the proportion of patients with severe pain was highest in the neuralgia group at 43% (CRPS: 27%; NLP: 37%; *p* = 0.026; see Figure 3).

**Table 2:** Outcome measures at follow‐up (T2).

Variable	*N*	CRPS *N* = 116	Neuralgia *N* = 68	NLP *N* = 75	*p*‐value
Mean pain intensity	259				0.005^#^
Mean ± SD		4.50 ± 2.60	5.77 ± 2.13	5.03 ± 2.77	
Median (Q1–Q3)		5 (3–7)	6 (4–7)	6 (3–7)	
Medication	259				0.347
No drug		39 (34%)	18 (26%)	17 (23%)	
1 or 2 drugs		41 (35%)	22 (32%)	32 (43%)	
3 or more drugs		36 (31%)	28 (41%)	26 (35%)	
Pain intensity and treatment	259				0.026^#^
No/low pain, no drugs		25 (22%)	5 (7.4%)	10 (13%)	
No/low pain under drugs		9 (7.8%)	3 (4.4%)	11 (15%)	
Moderate pain, no drugs		10 (8.6%)	10 (15%)	5 (6.7%)	
Moderate pain under drugs		41 (35%)	21 (31%)	21 (28%)	
Severe pain		31 (27%)	29 (43%)	28 (37%)	
Finger‐palm distance	163				< 0.001^+^
0 cm		37 (38%)	28 (67%)	19 (79%)	
0–1 cm		3 (3.1%)	2 (4.8%)	3 (13%)	
1–3 cm		26 (27%)	6 (14%)	1 (4.2%)	
> 3 cm		31 (32%)	6 (14%)	1 (4.2%)	
Thumb opposition	163				0.103
Full range of motion		66 (68%)	26 (62%)	22 (92%)	
Mildly impaired		18 (19%)	10 (24%)	2 (8.3%)	
Severely impaired		13 (13%)	6 (14%)	0 (0%)	
Use of aids (upper extremity)	163	32 (33%)	12 (29%)	9 (38%)	0.748
Treading	73				0.163
Good		0 (0%)	0 (0%)	0 (0%)	
Impaired		18 (100%)	22 (92%)	31 (100%)	
Impossible		0 (0%)	2 (8.3%)	0 (0%)	
Walking ability	85				0.245
Impossible		8 (44%)	9 (36%)	11 (26%)	
Few steps		3 (17%)	6 (24%)	3 (7.1%)	
> 100 m		3 (17%)	6 (24%)	15 (36%)	
> 1 km		4 (22%)	4 (16%)	13 (31%)	
Use of aids (lower extremity)	97	19 (95%)	20 (77%)	32 (63%)	0.020^+^
Functional outcome	255				< 0.001^+Δ^
Good		17 (15%)	5 (7.5%)	6 (8.3%)	
Moderate		17 (15%)	15 (22%)	31 (43%)	
Severe		82 (71%)	47 (70%)	35 (49%)	
Working ability	259				0.4
Good		52 (45%)	28 (41%)	40 (53%)	
Moderate		22 (19%)	16 (24%)	17 (23%)	
Severe		42 (36%)	24 (35%)	18 (24%)	
Global impression of disability	255				0.044^#^
None/slight		27 (23%)	6 (9.0%)	10 (14%)	
Moderate		32 (28%)	14 (21%)	19 (26%)	
Severe		57 (49%)	47 (70%)	43 (60%)	

*Note:* *p*‐values are calculated using Pearson’s *χ* ^2^‐test or Fisher’s exact test for categorical variables, and an ANOVA with Bonferroni‐corrected *t*‐tests for continuous variables. Significant post hoc tests between groups are displayed (CRPS—Neuralgia^#^; CRPS—NLP^+^; Neuralgia—NLP^Δ^).

### 3.3. Questionnaire and Outcome Measurements at Follow‐Up (T2)

#### 3.3.1. Functionality

At follow‐up (T2), significant differences in clinical outcomes were observed across the groups. Regarding upper extremity function, 67% of neuralgia and 79% of NLP patients demonstrated preserved fist closure, compared to only 38% of CRPS patients (*p* < 0.001; see Table 2). No significant differences were noted in thumb opposition or use of aids for the upper extremity. However, for the lower extremity, while treading and walking ability were comparable, the use of aids was significantly more common in the CRPS group (95%) compared to neuralgia (77%) and NLP (63%) patients (*p* = 0.020). Detailed information on aid use is provided in Table S2.

Functional outcomes differed significantly between the groups (*p* < 0.001). The CRPS group had the highest proportion of patients with severe impairment (71%) and a relatively low percentage with good outcomes (15%). In comparison, the NLP group had the highest proportion with moderate impairment (43%). NLP patients reported better functional outcomes compared to CRPS (*p* < 0.001) and neuralgia patients (*p* = 0.027; see Figure 3).

The global impression of disability also showed significant differences between the groups (*p* = 0.044). Neuralgia patients reported the highest percentage of severe disability (70%), while the CRPS group had the highest proportion in the “none/slight” disability category (23%).

#### 3.3.2. Severity of NLSs

In the analysis of NLSs, a significant difference in the distribution was observed for Item 1 (*p* = 0.033) and Item 3 (*p* = 0.016) among the three groups. Significant influence of mean pain intensity was observed across all items, while gender, age, and disease duration had only occasional influence on neglect‐like items. Post hoc analyses revealed a significant difference only between the CRPS and NLP groups for Item 3 (*p* = 0.036). The neglect‐like total score was 2.4 (2.2–2.6) in the CRPS group, 2.5 (2.3–2.7) in the neuralgia group, and 2.2. (1.9–2.5) in the NLP group (*p* = 0.055). The dichotomous items exhibited similar frequencies across all three groups. The number of patients who agreed to all items was comparable between groups (CRPS: 19.8%; neuralgia: 16.2%; NLP: 17.3%). Results are summarized in Table 3.

**Table 3:** Scoring and binary agreement of neglect‐like symptoms in CRPS, neuralgia, and nociceptive limb pain (NLP).

Item	Likert scale^a^			ANCOVA	Dichotomous scale^b^			Fisher’s test
	CRPS M (95%‐CI)	Neuralgia M (95%‐CI)	NLP M (95%‐CI)	*p*‐value^c^	CRPS agreement (%)	Neuralgia agreement (%)	NLP agreement (%)	*p*‐value
[1] *… without attention, it is like dead weight*	2.5 (2.2–2.8)	2.3 (2.0–2.7)	2.0 (1.7–2.4)	0.033	56.9	51.5	42.7	n.s.
[2] *… not part of my body*	2.4 (2.1–2.8)	2.6 (2.2–2.9)	2.2 (1.9–2.6)	0.564	52.6	58.8	45.3	n.s.
[3] *… focus attention to make it move*	3.2 (2.8–3.5)	3.4 (2.9–3.8)	2.8 (2.4–3.3)	0.016	64.7	70.6	54.7	n.s.
[4] *… moves without my control*	2.0 (1.7–2.2)	2.2 (1.8–2.5)	1.9 (1.6–2.2)	0.249	40.5	52.2	44.0	n.s.
[5] *… feels dead to me*	2.0 (1.7–2.2)	2.2 (1.9–2.5)	2.0 (1.6–2.3)	0.881	44.0	52.9	40.0	n.s.
Neglect‐like total score	2.4 (2.2–2.6)	2.5 (2.3–2.7)	2.2 (1.9–2.5)	0.055	19.8^d^	16.2^d^	17.3^d^	n.s.

^a^Values of the six‐option Likert scale (range: 1 = “never” up to 6 = “always”).

^b^Transformation of the six‐option Likert scale into the dichotomous categories (1 = “not true,” all other scale values 2–5 = “true”).

^c^Covariates: age, gender, disease duration, mean pain intensity.

^d^Agreement to all five items.

#### 3.3.3. Ability to Work

**Figure 4:**
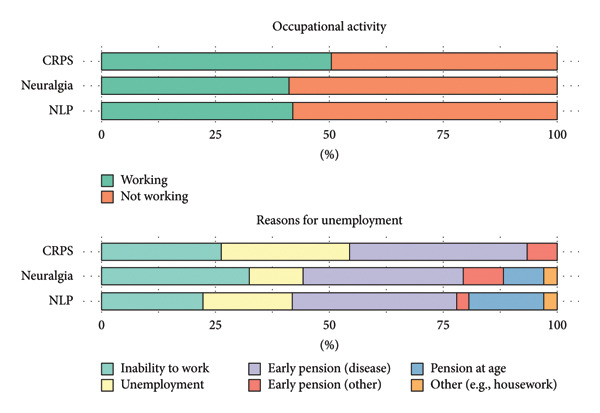
Overview of occupational activity and reasons for unemployment at follow‐up (T2).

Occupational activity at follow‐up (T2) was similar across patients with CRPS, NLP, and neuralgia. At T2, 51% of CRPS patients and 41% of neuralgia and NLP patients were employed (see Figure 4). There were no significant differences in working ability between the groups (*p* = 0.397). Good working ability, defined by the proportion of time to work relative to the time since disease onset, was achieved by 45% of CRPS, 41% of neuralgia, and 53% of NLP patients.

The main reasons for unemployment were inability to work (CRPS: 26%; NLP: 22%; neuralgia: 32%), unemployment (CRPS: 28%; NLP: 19%; neuralgia: 12%), and early retirement due to disease (CRPS: 39%; NLP: 36%; neuralgia: 35%). Retirement at the expected age during the study period was reported by 17% of NLP patients, 9% of neuralgia patients, and none of the CRPS group.

#### 3.3.4. Predictors for Outcome in Study Groups

Multivariate logistic regression analysis revealed distinct predictors of outcome across study groups. CPRS patients without occupation at T0 faced a lower risk of a severe outcome than full‐time workers (OR = 0.2; 95% CI: 0.1–0.8; *p* = 0.029). In the NLP, part‐time workers had a lower risk of severe outcome than full‐time workers (OR = 0.012; 95% CI: 0.0003–0.3; *p* = 0.0135), and higher pain at T0 was linked to increased risk of a severe outcome (OR 1.9; 95% CI: 1.2–3.2; *p* = 0.013). For neuralgia patients, a disease duration of 1–3 years significantly elevated the risk of poor outcome compared to patients in the first 90 days after diagnosis (OR 18.6; 95%‐CI: 1.1–442.6; *p* = 0.048), with a similar trend observed for durations ≥ 3 years (OR 10.5; 95%‐CI: 0.8–186.3; *p* = 0.082). In CRPS, the number of positive clinical signs was a strong predictor: CRPS patients with three (OR = 6.4; 95% CI: 1.3–38.6; *p* = 0.027) and four signs (OR = 6.3; 95%‐CI: 1.4–37.8; *p* = 0.027) exhibited a higher risk compared to patients with only two signs.

#### 3.3.5. Subgroup Analysis of Cortisone Therapy in CRPS Patients

Subgroup analysis of CRPS patients treated with corticosteroids revealed that those selected for this therapy exhibited more positive clinical signs and experienced less favorable outcomes than patients who did not require corticosteroid treatment (see Table S3). However, pain intensity at T0 (5.58 ± 1.93 vs. 5.19 ± 2.17) and T2 (4.95 ± 2.39 vs. 4.05 ± 2.75) did not differ significantly between the groups (*p* ≥ 0.059). Both groups improved functional status over time (*p* ≤ 0.010; see Table S4). Patients not treated with corticosteroids showed a significant improvement in pain intensity over time (*p* = 0.006), whereas those receiving corticosteroids did not exhibit a statistically significant change (*p* = 0.093).

#### 3.3.6. Interrelationship Between Main Clinical Outcomes

**Figure 5:**
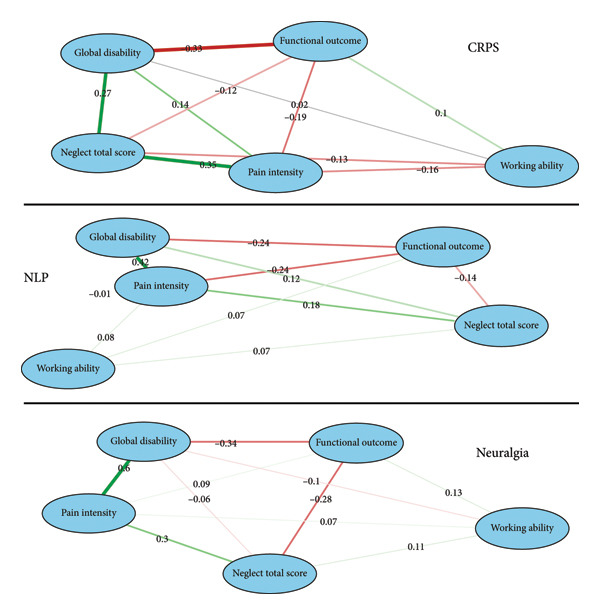
Exploratory network models illustrating the interrelationships among key clinical variables across diagnostic groups. Separate networks were estimated for each group using partial correlation analysis (qgraph package, Version 1.9.8, R), with each edge representing the unique association between two variables after controlling for all others. Edge thickness is proportional to the magnitude of the partial correlation, such that thicker edges indicate stronger associations. Edge color denotes the direction of the partial correlation (red—positive associations; red—negative associations). Node size is held constant across all variables and therefore does not convey statistical information. Node positions reflect a force‐directed layout influenced by the pattern of partial correlations, which places more strongly interconnected variables closer to the center and weakly connected variables toward the periphery, thereby facilitating visual interpretation of network structure. Together, these visual features highlight differences in connectivity patterns across diagnostic groups.

Partial correlation networks revealed distinct patterns across diagnostic groups. A consistent core structure was observed in all groups, characterized by interconnections between pain intensity, functional outcome, and global disability (see Figure 5). Pain and function were tightly linked to perceived disability. In the CRPS group, Neglect Total Score showed pronounced positive associations with both pain intensity (*r* = 0.35) and global disability (*r* = 0.27), along with a negative association with functional outcome (*r* = −0.19). These relationships were notably weaker or absent in the NLP and neuralgia groups, suggesting a diagnosis‐specific role of perceptual neglect in CRPS. Across all groups, working ability consistently exhibited the weakest direct associations (*r* ≤ 0.16).

## 4. Discussion

This study aimed to evaluate long‐term outcomes in patients with CRPS in comparison with patients suffering from neuralgia NLP. Special attention was given to work‐related disability, predictors of functional outcomes, and the frequency of NLSs.

### 4.1. Comparison of Clinical Data and Pain Intensity Between Study Groups

Although all groups shared similar demographic characteristics, the recruited CRPS patients were referred to the pain clinic earlier in their disease course. Nevertheless, the mean disease duration at the time of referral was approximately 6 months, substantially longer than the timeframe of 2 months suggested in current clinical guidelines [19]. Similarly, a recent analysis of claims data reported that real‐world treatment of CRPS does not completely reflect recommendations in clinical practice guidelines [20]. As expected, CRPS patients exhibited more positive clinical signs at baseline, particularly vasomotor and sudomotor disturbances, consistent with the Budapest criteria and the typical clinical presentation of CRPS. Despite the shorter disease duration, mean pain intensity in the CRPS cohort was comparable to that of the control groups. This suggests that pain levels in CRPS are comparable to those in chronic extremity pain of other origins, even when present over years. Over time, pain intensity significantly declined in both the CRPS and NLP groups, while it remained unchanged in patients with neuralgia. Interestingly, patients with CRPS exhibited a mild secondary worsening of symptoms, which may reflect, on the one hand, a transient effect of corticosteroid treatment administered between baseline and the first clinical follow‐up (T1), and on the other hand, a transition toward centrally mediated mechanisms such as central sensitization, which has been described as contributing to the persistence and amplification of pain in CRPS patients [21, 22]. On the other side, the plateau in the neuralgia group may reflect fully exhausted treatment options already at baseline compared to CRPS. Interestingly, another survey also reported that individuals with CRPS Type 1 more than CRPS Type 2 answered that their disability was reduced over time, indicating the impact of a concomitant nerve injury on ongoing disability [23]. The differences compared to NLP may be attributed to the distinct pathophysiological mechanisms underlying neuropathic pain, in contrast to nociceptive pain. This is supported by the fact that medication usage became comparable among the groups by the end of the study. Notably, a considerable proportion of CRPS patients (22%) reported low pain without medication, highlighting the potential for improvement in some cases and underscoring the heterogeneity of the disease course.

### 4.2. Functionality

The follow‐up data revealed substantial functional deficits in all study groups. While fist closure was preserved in the majority of NLP and neuralgia patients, only a minority of CRPS patients retained this ability, underscoring the marked impairment associated with CRPS. Interestingly, no significant differences were observed in thumb opposition or upper limb aid usage, suggesting that certain fine motor functions may be less discriminative across conditions. A possible explanation is the alterations in bone metabolism commonly associated with the condition. Imaging studies have shown increased osteoclastic and osteoblastic activity, leading to localized osteoporosis, elevated bone turnover, and inflammatory bone remodeling [24]. These changes predominantly affect the metacarpophalangeal and interphalangeal joints, resulting in joint stiffness and reduced mobility, which are critical for effective finger flexion [25]. This may account for the greater limitation in fist closure compared to thumb opposition, particularly when contrasted with other pain groups. In contrast, the reliance on assistive devices for lower extremity function was notably higher in the CRPS group, despite similar walking and tread abilities, potentially reflecting a greater perceived or experienced disability [26].

While CRPS and neuralgia patients exhibited more pronounced functional impairments compared to those with NLP, only about half of the CRPS patients reported a severe global impression of disability. In contrast, this perception was present in 70% of neuralgia patients. Since exploratory network analysis indicated that the global impression of disability is particularly influenced by functional outcome and pain intensity, this discrepancy may partly reflect the lower pain intensity reported by CRPS patients relative to those with neuralgia. These results underscore the heterogeneous nature of functional limitations across different chronic pain conditions and suggest that a satisfactory outcome remains achievable in CRPS, despite its often severe presentation.

### 4.3. NLSs

NLSs, characterized by altered perception of the affected limb, can occur in chronic limb pain but are typically more prevalent in CRPS [17, 27]. Their presence has been associated with worse pain outcomes in CRPS patients after 6 months [28]. In our study, significant group differences were found in the frequency distribution of Items 1 and 3, both of which are indicative of motor neglect. However, post hoc analyses revealed a significant difference only between CRPS and NLP in Item 3, while no other pairwise differences reached statistical significance. This is surprising, as prior studies consistently report rates of all NLS items among CRPS patients compared to other chronic limb pain populations [17, 18, 28–30]. In our cohort, the lack of more pronounced group differences seems to be driven by a slightly lower prevalence in the CRPS groups, and more notably, by an unexpectedly high prevalence in the neuropathy group. In line with previous studies, we found no significant influence of disease duration and demographic factors, such as age and gender, on NLS [17, 28, 29]. In contrast, pain intensity significantly influenced NLS severity, suggesting a central role of nociceptive processing in the manifestation of these symptoms. Given that the neuropathy group in our study reported significantly higher pain scores than the CRPS group, this may explain the high prevalence of NLS within that cohort. This is supported by the strong association between NLS and pain intensity across all study groups observed in the exploratory network analysis. Notably, the pronounced positive associations of NLS with both pain intensity and global impression of disability in the CRPS group underline its relevance in this condition. These findings could carry clinical implications. Interventions targeting central mechanisms—such as graded motor imagery, mirror therapy, or other approaches aimed at restoring distorted body representation—may benefit not only CRPS patients but also individuals with high‐intensity neuropathic pain [31]. This underscores the importance of routinely assessing body perception disturbances in chronic pain management, beyond CRPS alone.

### 4.4. Working Ability and Reasons for Work Inactivity

While previous studies reported unemployment rates among CRPS patients ranging from 30% to 80%, approximately half of the CRPS patients in our cohort were employed at follow‐up [32–34]. Notably, work‐related disability emerged as a major long‐term burden across all groups, with comparable rates of employment impairment, despite CRPS and neuralgia patients exhibiting more pronounced functional limitations than those with NLP. Furthermore, the consistently weak associations between working ability and pain intensity, functional outcome, NLS, and global impression of disability across all study groups in the network analysis suggest limited explanatory value in this context. This finding suggests that occupational status may not be determined solely by clinical severity but is likely influenced by a complex interplay of psychosocial and socioeconomic factors [4]. Recent literature has identified low functional capacity, high biopsychosocial complexity, severe injury, lack of formal employment contracts, and lower educational attainment as key predictors of long‐term work incapacity in CRPS [26, 35, 36]. In our study, the most common reason for not working across all groups was early retirement due to disease, underlining the substantial long‐term impact of chronic pain on occupational functioning.

### 4.5. Predictors of Global Disability

It is not surprising that CRPS patients presenting with a higher number of positive clinical signs at the initial examination are at increased risk for more severe functional impairment, which aligns with observations from routine clinical practice [37]. Interestingly, CRPS patients without a prior occupation had a lower risk of poor outcome than full‐time workers. A similar association was observed for part‐time employment in the NLP group. This counterintuitive result might reflect differences in baseline physical demands or psychosocial stressors related to job loss and return‐to‐work pressure [5]. Additionally, the association may be influenced by reverse causation—meaning that individuals with less functional impairment are more likely to resume work, rather than occupational activity itself contributing to symptom exacerbation. While high baseline pain intensity predicted an unfavorable prognosis in NLP patients, for neuralgia, the early phase after diagnosis appears to be particularly favorable and critical for treatment. This highlights the complex interplay between employment status and clinical outcome and underscores the need for longitudinal data to clarify the directionality of this relationship.

Subgroup analysis of corticosteroid treatment showed that this therapy was more frequently administered to patients with more severe clinical signs. While both groups demonstrated functional improvement over time, the lack of significant pain reduction in the corticosteroid‐treated group, unlike in the less affected group, suggests that corticosteroids may support certain aspects of recovery. However, their effect on pain remains uncertain [29, 30]. These findings underscore the need for controlled studies to enhance the quality and standardization of CRPS treatment [28].

In general, several mechanisms may have contributed to the observed improvement in our cohort. In patients with CRPS, the alignment of pharmacotherapy with that of the comparison groups may have played a role—particularly through the addition of medications targeting neuropathic pain, such as gabapentinoids or antidepressants. Furthermore, a subset of patients underwent multimodal inpatient pain therapy between T0 and T1; however, this was unfortunately not systematically documented. Interestingly, despite the long duration since diagnosis in both NLP and neuralgia groups, patients with NLP appeared to benefit from treatment in a specialized pain clinic, whereas this effect less pronounced in patients with longstanding neuralgia, at least with respect to pain outcomes. One possible explanation is that patients with NLP may retain a degree of central or peripheral plasticity that renders them more responsive to interdisciplinary interventions, whereas chronic neuralgia may involve more entrenched mechanisms that are less amenable to change. Additionally, differences in psychosocial burden, coping strategies, or prior treatment history might have influenced responsiveness to therapy.

### 4.6. Limitations

Although this study offers a comprehensive overview of functional outcomes in CRPS compared to two demographically similar control groups, several limitations should be acknowledged. First, the group sizes differed notably, influencing statistical power and group comparisons. Although machine‐learning approaches were considered to explore potential nonlinearities and interaction effects, simpler statistical methods were chosen due to sample size constraints and the need for interpretable parameter estimates. Second, the disease duration varied significantly between the groups, potentially affecting symptom severity, functional outcomes, and perception of disability. Third, the follow‐up was conducted via telephone survey, which, while practical, may limit the depth and objectivity of clinical assessments compared to in‐person evaluations. Furthermore, no standardized clinical examinations were conducted longitudinally, restricting the ability to draw conclusions about changes in objective clinical signs over time. Additionally, the lack of systematic data on treatments received (like inpatient multimodal therapy) is a constraint on identifying the drivers of improvement. Finally, in the context of the CRPS cohort, it must be acknowledged that the inclusion criterion of a minimum of 1 year of pain treatment likely excluded patients with acute or transient disease courses. As a result, the sample predominantly reflects individuals with more persistent and potentially treatment‐resistant symptomatology. This selection may contribute to an overall more severe clinical profile and functional outcome compared to the broader CRPS population, thereby limiting the generalizability of findings to early‐stage or less chronic cases.

## 5. Conclusion

Despite shorter disease duration, CRPS patients exhibited similar pain intensity to neuralgia and NLP patients, and more pronounced clinical signs. Nevertheless, the global impression of disability in CRPS was not necessarily worse than in control groups, suggesting that the overall outcome in CRPS is comparable to long‐lasting limb pain. The comparable work‐related disability across all groups also reflects this. Finally, corticosteroid therapy may support functional improvement in more severely affected CRPS patients, though its analgesic effects remain unclear. Overall, the findings emphasize the heterogeneous course of CRPS and underline the importance of individualized, multidimensional assessment and treatment strategies.

## Conflicts of Interest

The authors declare no conflicts of interest.

## Author Contributions

Johannes Forsting and Elena Enax‐Krumova contributed equally to this study.

## Funding

The authors received no specific funding for the project. Open Access funding enabled and organized by Projekt DEAL.

## Supporting Information

Supporting Table 1: Overview of pharmacotherapy over time and interventional treatments.

Supporting Table 2: Overview of clinical data and outcome measures in CRPS patients with and without corticosteroid treatment.

Supporting Table 3: Overview of clinical data and outcome measures in CRPS patients with and without corticosteroid treatment.

Supporting Table 4: Overview of finger‐palm distance, pain intensity, and treatment over time in CRPS patients with and without corticosteroid treatment.

Supporting Clinical Report Form including the used Questionnaires (in German).

## Supporting information


**Supporting Information** Additional supporting information can be found online in the Supporting Information section.

## Data Availability

Data are available from the corresponding author upon reasonable request.
