# Supplementary material for: Long‐Term Outcome of Complex Regional Pain Syndrome versus Limb Pain of Other Origin: Results From a Telephone Survey With up to 5‐year Follow‐Up
Source: Pain Res Manag. 2026 Jan 21;2026:4722836. doi: 10.1155/prm/4722836 (PMC12823771; doi:10.1155/prm/4722836)
Supplement: Supplementary file 1 — Supporting Information Additional supporting information can be found online in the Supporting Information section. [file PRM-2026-4722836-s001.zip › Arslan2025_CRPSNeuralgiaNLP_Supplementary.docx]

Table S1: Overview of pharmacotherapy over time and interventional treatments.

| **Variable** | **N** | **CRPS**  N = 116 | **Neuralgia**  N = 68 | **NLP**  N = 75 | **p-value** |
| --- | --- | --- | --- | --- | --- |
| **Medication at T0** | 256 |  |  |  | 0.004*^#+^* |
| no drug |  | 22 (19%) | 9 (13%) | 9 (12%) |  |
| 1 or 2 drugs |  | 75 (66%) | 33 (49%) | 42 (56%) |  |
| 3 or more drugs |  | 16 (14%) | 26 (38%) | 24 (32%) |  |
| **Medication at T1** | 259 |  |  |  | <0.001^+^*^∆^* |
| no drug |  | 35 (30%) | 5 (7.4%) | 15 (20%) |  |
| 1 or 2 drugs |  | 56 (48%) | 33 (49%) | 40 (53%) |  |
| 3 or more drugs |  | 25 (22%) | 30 (44%) | 20 (27%) |  |
| **Medication at T2** | 259 |  |  |  | 0.3 |
| no drug |  | 39 (34%) | 18 (26%) | 17 (23%) |  |
| 1 or 2 drugs |  | 41 (35%) | 22 (32%) | 32 (43%) |  |
| 3 or more drugs |  | 36 (31%) | 28 (41%) | 26 (35%) |  |
| **NSAID at T0** | 258 | 61 (53%) | 36 (53%) | 47 (64%) | 0.3 |
| **NSAID at T1** | 257 | 26 (23%) | 32 (47%) | 25 (34%) | 0.003^#^ |
| **NSAID at T2** | 259 | 19 (16%) | 10 (15%) | 27 (36%) | 0.002^+^*^∆^* |
| **Opioids at T0** | 258 | 21 (18%) | 18 (26%) | 20 (27%) | 0.3 |
| **Opioids at T1** | 257 | 15 (13%) | 21 (31%) | 18 (24%) | 0.012^#^ |
| **Opioids at T2** | 259 | 19 (16%) | 26 (38%) | 16 (21%) | 0.003^#^ |
| **Antidepressants at T0** | 258 | 9 (7.8%) | 17 (25%) | 15 (20%) | 0.004^#+^ |
| **Antidepressants at T1** | 257 | 24 (21%) | 23 (34%) | 18 (24%) | 0.15 |
| **Antidepressants at T2** | 259 | 19 (16%) | 10 (15%) | 5 (6.7%) | 0.14 |
| **TCA at T0** | 257 | 7 (6.0%) | 11 (16%) | 10 (14%) | 0.066 |
| **TCA at T1** | 256 | 20 (18%) | 12 (18%) | 11 (15%) | 0.9 |
| **TCA at T2** | 259 | 14 (12%) | 6 (8.8%) | 4 (5.3%) | 0.3 |
| **SSRI at T0** | 257 | 1 (0.9%) | 4 (6.0%) | 6 (8.1%) | 0.040^+^ |
| **SSRI at T1** | 256 | 2 (1.8%) | 8 (12%) | 5 (6.8%) | 0.019^#^ |
| **SSRI at T2** | 259 | 1 (0.9%) | 0 (0%) | 0 (0%) | 0.5 |
| **SNRI at T0** | 258 | 1 (0.9%) | 4 (5.9%) | 3 (4.1%) | 0.14 |
| **SNRI at T1** | 256 | 4 (3.5%) | 3 (4.4%) | 4 (5.4%) | 0.8 |
| **SNRI at T2** | 259 | 4 (3.4%) | 4 (5.9%) | 0 (0%) | 0.12 |
| **Anticonvulsant at T0** | 258 | 20 (17%) | 22 (32%) | 12 (16%) | 0.026^#^*^∆^* |
| **Anticonvulsant at T1** | 257 | 19 (17%) | 23 (34%) | 18 (24%) | 0.027^#^ |
| **Anticonvulsant at T2** | 259 | 19 (16%) | 15 (22%) | 12 (16%) | 0.6 |
| **Metamizol at T0** | 258 | 40 (34%) | 24 (35%) | 27 (36%) | >0.9 |
| **Metamizol at T1** | 256 | 56 (49%) | 36 (53%) | 40 (55%) | 0.7 |
| **Metamizol at T2** | 259 | 54 (47%) | 32 (47%) | 30 (40%) | 0.6 |
| **Botox** | 258 | 7 (6.0%) | 14 (21%) | 4 (5.4%) | 0.002^#^*^∆^* |
| **Sympathetic blockade** | 259 | 9 (7.8%) | 3 (4.4%) | 2 (2.7%) | 0.3 |
| **SCS/PNS** | 259 | 0 (0%) | 3 (4.4%) | 0 (0%) | 0.014 |
| *p-values are calculated using Pearson’s χ^2^-test or Fisher’s exact test. Significant post-hoc tests between groups are displayed (CRPS – Neuralgia ^#^; CRPS – NLP ^+^; Neuralgia – NLP ^∆^).* | | | | | |
|  | | | | | |

Table S2: Overview of clinical data and outcome measures in CRPS patients with and without corticosteroid treatment.

| **Variable** | **N** | **CRPS**  N = 116 | **Neuralgia**  N = 68 | **NLP**  N = 75 | **p-value** |
| --- | --- | --- | --- | --- | --- |
| **Use of aids (UE)** | 163 | 32 (33%) | 12 (29%) | 9 (38%) | 0.7 |
| **Orthosis** | 53 | 16 (50%) | 5 (42%) | 6 (67%) | 0.5 |
| **Splint** | 52 | 19 (59%) | 2 (18%) | 2 (22%) | 0.021 |
| **Others** | 52 | 8 (25%) | 6 (55%) | 3 (33%) | 0.2 |
| **Use of aids (LE)** | 97 | 19 (95%) | 20 (77%) | 32 (63%) | 0.020*^∆^* |
| **Wheelchair** | 71 | 0 (0%) | 2 (10%) | 1 (3.1%) | 0.3 |
| **Rollator** | 71 | 0 (0%) | 2 (10%) | 3 (9.4%) | 0.4 |
| **Crutch** | 71 | 3 (16%) | 3 (15%) | 6 (19%) | >0.9 |
| **Crutches** | 71 | 3 (16%) | 3 (15%) | 8 (25%) | 0.6 |
| **Orthosis** | 71 | 3 (16%) | 8 (40%) | 12 (38%) | 0.2 |
| **Orthopedic shoes** | 71 | 15 (79%) | 9 (45%) | 17 (53%) | 0.078 |
| *p-values are calculated using Pearson’s χ^2^-test or Fisher’s exact test. Significant post-hoc tests between groups are displayed (CRPS – Neuralgia ^#^; CRPS – NLP ^+^; Neuralgia – NLP ^∆^).* | | | | | |

Table S3: Overview of clinical data and outcome measures in CRPS patients with and without corticosteroid treatment.

| **Variable** | **N** | **Cortison -**  N = 61 | **Cortison +**  N = 55 | **p-value** |
| --- | --- | --- | --- | --- |
| **Age** | 116 |  |  | 0.039 |
| Mean ± SD |  | 57 ± 13 | 52 ± 11 |  |
| Median (Q1–Q3) |  | 58 (50–65) | 53 (47–60) |  |
| **Gender** | 116 |  |  | 0.6 |
| female |  | 35 (57%) | 28 (51%) |  |
| male |  | 26 (43%) | 27 (49%) |  |
| **BMI** | 114 |  |  | 0.2 |
| Mean ± SD |  | 26.2 ± 4.9 | 27.5 ± 5.1 |  |
| Median (Q1–Q3) |  | 25.5 (22.5–28.7) | 27.2 (24.6–30.1) |  |
| **Initiating factor** | 116 |  |  | 0.9 |
| acute trauma |  | 54 (89%) | 50 (91%) |  |
| posttraumatic alteration |  | 2 (3.3%) | 1 (1.8%) |  |
| other disease |  | 5 (8.2%) | 4 (7.3%) |  |
| **Disease duration** | 116 |  |  | 0.2 |
| < 90d |  | 8 (13%) | 13 (24%) |  |
| 90–179d |  | 26 (43%) | 27 (49%) |  |
| 180–365d |  | 16 (26%) | 9 (16%) |  |
| 1–3y |  | 11 (18%) | 5 (9.1%) |  |
| > 3y |  | 0 (0%) | 1 (1.8%) |  |
| **Mean pain intensity at T0** | 116 |  |  | 0.4 |
| Mean ± SD |  | 5.26 ± 2.16 | 5.58 ± 1.93 |  |
| Median (Q1–Q3) |  | 5 (4–7) | 5 (4–7) |  |
| **Medication at T0** | 113 |  |  | 0.2 |
| no drug |  | 15 (25%) | 7 (13%) |  |
| 1 or 2 drugs |  | 36 (61%) | 39 (72%) |  |
| 3 or more drugs |  | 8 (14%) | 8 (15%) |  |
| **Positive clinical signs** | 116 |  |  | <0.001 |
| 2 |  | 17 (28%) | 1 (1.8%) |  |
| 3 |  | 16 (26%) | 16 (29%) |  |
| 4 |  | 28 (46%) | 38 (69%) |  |
| **Sensory signs** | 116 | 49 (80%) | 51 (93%) | 0.10 |
| **Vasomotor signs** | 116 | 39 (64%) | 45 (82%) | 0.052 |
| **Sudomotor signs** | 116 | 45 (74%) | 51 (93%) | 0.014 |
| **Motor/trophic signs** | 116 | 61 (100%) | 55 (100%) |  |
| **Mean pain intensity at T2** | 116 |  |  | 0.077 |
| Mean ± SD |  | 4.10 ± 2.73 | 4.95 ± 2.39 |  |
| Median (Q1–Q3) |  | 4.5 (2–6) | 5 (4–7) |  |
| **Pain intensity and treatment** | 116 |  |  | 0.3 |
| no/low pain, no drugs |  | 18 (30%) | 7 (13%) |  |
| no/low pain under drugs |  | 4 (6.6%) | 5 (9.1%) |  |
| moderate pain, no drugs |  | 5 (8.2%) | 5 (9.1%) |  |
| moderate pain under drugs |  | 20 (33%) | 21 (38%) |  |
| severe pain |  | 14 (23%) | 17 (31%) |  |
| **Functional outcome** | 116 |  |  | 0.014 |
| good |  | 12 (20%) | 5 (9.1%) |  |
| moderate |  | 13 (21%) | 4 (7.3%) |  |
| severe |  | 36 (59%) | 46 (84%) |  |
| **Working ability** | 116 |  |  | 0.016 |
| good |  | 34 (56%) | 18 (33%) |  |
| moderate |  | 12 (20%) | 10 (18%) |  |
| severe |  | 15 (25%) | 27 (49%) |  |
| **Global impression of disability** | 116 |  |  | 0.003 |
| none/slight |  | 17 (28%) | 10 (18%) |  |
| moderate |  | 23 (38%) | 9 (16%) |  |
| severe |  | 21 (34%) | 36 (65%) |  |
| *p-values are calculated using Pearson’s χ^2^-test or Fisher’s exact test for categorical variables, and t-tests for continuous variables.* | | | | |

|  |
| --- |

Table S4: Overview of finger-palm distance, pain intensity and treatment over time in CRPS patients with and without corticosteroid treatment.

| Group | **Variable** | **T0** | **T2** | **p-value** |
| --- | --- | --- | --- | --- |
|  | **Finger-palm distance** | N = 47 | | 0.010 |
| Cortison + | 0cm | 4 (8%) | 12 (26%) |  |
|  | < 1cm | 4 (8%) | 0 (0%) |  |
|  | 1–3cm | 12 (26%) | 13 (28%) |  |
|  | > 3cm | 27 (58%) | 22 (47%) |  |
|  | **Finger-palm distance** | N = 50 | | 0.004 |
| Cortison - | 0cm | 12 (24%) | 25 (50%) |  |
|  | < 1cm | 10 (20%) | 3 (6%) |  |
|  | 1–3cm | 10 (20%) | 13 (26%) |  |
|  | > 3cm | 18 (36%) | 9 (18%) |  |
|  | **Pain intensity** | N = 55 | | 0.093 |
| Cortison + | None | 0 (0%) | 6 (11%) |  |
|  | Low | 9 (16%) | 6 (11%) |  |
|  | Moderate | 28 (51%) | 26 (47%) |  |
|  | Severe | 18 (33%) | 17 (31%) |  |
|  | **Pain intensity** | N = 61 | | 0.006 |
| Cortison - | None | 2 (3%) | 13 (21%) |  |
|  | Low | 11 (18%) | 9 (15%) |  |
|  | Moderate | 29 (48%) | 25 (41%) |  |
|  | Severe | 19 (31%) | 14 (23%) |  |

*p-values were calculated using the Stuart-Maxwell test.*
