# Supplementary material for: Long‐Term Outcome of Complex Regional Pain Syndrome versus Limb Pain of Other Origin: Results From a Telephone Survey With up to 5‐year Follow‐Up
Source: Pain Res Manag. 2026 Jan 21;2026:4722836. doi: 10.1155/prm/4722836 (PMC12823771; doi:10.1155/prm/4722836)
Supplement: Supplementary file 1 — Supporting Information Additional supporting information can be found online in the Supporting Information section. [file PRM-2026-4722836-s001.zip › Supplement_Questionnaires.pdf]

CRPS-Nachbefragung*

Wir hatten Sie vor kurzem schriftlich über dieses Telefoninterview informiert. Sie waren aufgrund Ihrer *CRPS-Erkrankung bzw. Neuralgie/ posttraumatischen Gelenkschmerzen* bei uns in der Schmerzmedizin in Behandlung. Wir möchten nun gerne einen Überblick darüber bekommen, wie der Krankheitsverlauf bei Ihnen bis heute verlief. Dabei ist uns insbesondere Ihr Befinden hinsichtlich der Schmerzen, der Gebrauchsfähigkeit im alltäglichen Leben, sowie Ihre derzeitige Arbeitsfähigkeit wichtig, damit wir in Zukunft Patienten besser beraten können und die Behandlung des CRPS optimieren können.
Das Interview dauert ca. 25 Minuten.

1. **Sind Sie einverstanden heute oder später an dem Telefoninterview teilzunehmen?**
   ☐ ja ☐ nein

Wenn nein: ***Vielen Dank. Alles Gute!***
Wenn ja: ***Bevor ich zum Thema Schmerzen komme, eine ganz andere Frage zuerst:***

2. **Sind Sie Links- oder Rechtshänder? / Mit welchem Bein haben Sie früher einen Ball geschossen?**
   ☐ links ☐ rechts

Zunächst würde Ich Sie gerne nach den Schmerzen befragen.

3. **Haben Sie heute oder in der letzten Woche noch Schmerzen *an der Hand/ an dem Fuß?***
   ☐ ja
   Wenn ja: **Wenn Sie sich eine Skala von 0 bis 10 vorstellen, wobei 0 gar keine und 10 die stärkten Schmerzen darstellen, die Sie sich vorstellen können, wie stark waren Ihre Schmerzen in der letzten Woche im Mittel?**
   ➔ Skala NRS:
   Wann treten diese Schmerzen auf? *(Mehrfachnennung möglich)*
   ☐ in Ruhe
   ☐ bei Belastung
   ☐ schon bei Alltagsbelastung
   ☐ erst bei stärkeren, z.B. beruflichen Tätigkeiten (z.B. Treppe steigen, Gewichte heben)
   ☐ nein

4. **Nehmen Sie heute oder in der letzten Woche noch wegen dieser Schmerzen Schmerzmedikamente ein?**
   ☐ nein **Seit wann benutzen Sie keine Schmerzmedikamente mehr?**
   Seit ____________________ Jahre
   ☐ ja, wegen dieser Hand-/Arm- bzw. Bein-/Fußschmerzen
   ☐ ja, aber wegen anderen Schmerzen

   Welche Schmerzmedikamente nehmen Sie ein?
   ____

   Wie oft nehmen sie die Medikamente ein?
   ☐ fest verordnet
   ☐ bei Bedarf

5. Beim CRPS der oberen Extremität: Nun frage ich nach der Funktionsfähigkeit Ihrer betroffenen Hand. Die Fragen beziehen sich auf Ihr Befinden heute oder in der letzten Woche
Bitte vergleichen Sie Ihre Hand mit dem Zustand vor der Erkrankung.

5.1 Brauchen Sie dabei Hilfsmittel? *(Mehrfachnennung möglich)*

☐ ja → ☐ Stabilisierungsprothesen(z.B. *Handgelenksmanschette, Orthese)*
☐ Ruheschiene
☐ Hilfsmittel für Besteck und Ähnliches
☐ nein

5.2 Können Sie jetzt die erkrankte Hand zur Faust schließen?

☐ ja, alle Finger
☐ ja, außer: **Rechts** **Links**

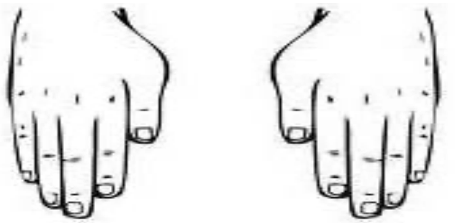

→ **Woran liegt das?** *(Mehrfachnennung möglich)*
☐ Steifigkeit ☐ schlechte Beweglichkeit
☐ Schmerzen ☐ Kraft

Wenn ja: **Sind dabei Ihre Fingernägel sichtbar?**
→ ☐ **ja** *(inkompletter Faustschluss)*

→ ☐ **nein** *(kompletter Faustschluss)*
☐ nein
→ **Woran liegt das?** *(Mehrfachnennung möglich)*
☐ Steifigkeit ☐ schlechte Beweglichkeit
☐ Schmerzen ☐ Kraft

5.3 Wenn Sie nun diese Finger auslassen und die beweglichen Finger betrachten, können Sie einschätzen, wie groß der Abstand der Fingerspitzen zu Ihrem Handballen beträgt? Sie können sich hierfür den beigefügten Maßstab zur Hilfe nehmen, der im Brief aufgedruckt wurde/ Wie viele Finger passen zwischen Handfläche und Finger?

FHA:__________cm

Fingeranzahl	Mann	Frau
1	<3cm	<2cm
2	4-5cm	3-4cm
3	6-7cm	5-6cm
4	>7cm	>6cm

5.4 Nehmen Sie den Daumen Ihrer betroffenen Seite und berühren Sie mit diesem Ihre Fingerspitzen. Bis zu welchem Finger können Sie mit dem Daumenballen die Fingerspitzen berühren?
Digitus: _______

5.5 Wie gut können Sie sich mit der erkrankten Hand über den Hinterkopf streichen?

☐ Kann ich gut.
☐ Kann ich eingeschränkt.
☐ Kann ich gar nicht.

Wenn eingeschränkt oder gar nicht möglich:
Was sind die Gründe hierfür? *(Mehrfachnennung möglich)*
☐ Schmerzen ☐ Kraft
☐ Beweglichkeit ☐ Keine Bewegungskontrolle möglich

5.6 Wie gut können Sie mit der erkrankten Hand Besteck benutzen?

☐ Kann ich gut.
☐ Kann ich eingeschränkt.
☐ Kann ich gar nicht.

Wenn eingeschränkt oder gar nicht möglich:
Was sind die Gründe hierfür? *(Mehrfachnennung möglich)*
☐ Schmerzen ☐ Kraft
☐ Beweglichkeit ☐ Keine Bewegungskontrolle möglich

5.7 Wie gut können Sie mit Ihrer erkrankten Hand eine Tasse hochheben?

☐ Kann ich gut.
☐ Kann ich eingeschränkt.
☐ Kann ich gar nicht.

Wenn eingeschränkt oder gar nicht möglich:
Was sind die Gründe hierfür? *(Mehrfachnennung möglich)*
☐ Schmerzen ☐ Kraft
☐ Beweglichkeit ☐ Keine Bewegungskontrolle möglich

5.8 Wie gut können Sie mit Ihrer erkrankten Hand eine Tasse absetzen?

☐ Kann ich gut.
☐ Kann ich eingeschränkt.
☐ Kann ich gar nicht.

Wenn eingeschränkt oder gar nicht möglich:
Was sind die Gründe hierfür? *(Mehrfachnennung möglich)*
☐ Schmerzen ☐ Kraft
☐ Beweglichkeit ☐ Keine Bewegungskontrolle möglich

5.9 Wie gut können Sie mit der betroffenen Hand den Schraubverschluss einer Flasche öffnen?

☐ Kann ich gut.
☐ Kann ich eingeschränkt.
☐ Kann ich gar nicht.

Wenn eingeschränkt oder gar nicht möglich:
Was sind die Gründe hierfür? *(Mehrfachnennung möglich)*
☐ Schmerzen ☐ Kraft
☐ Beweglichkeit ☐ Keine Bewegungskontrolle möglich

5.10 Wie stark sind Sie abschließend betrachtet bei der Benutzung Ihrer erkrankten Hand im Vergleich zum Zustand vor der Erkrankung beeinträchtigt?
☐ gar nicht beeinträchtigt
☐ wenig beeinträchtigt
☐ mittelmäßig beeinträchtigt
☐ stark beeinträchtigt

5.11 Die folgenden Aussagen betreffen die Wahrnehmung der erkrankten Hand. Bei manchen Patienten entsteht ein verändertes Gefühl der erkrankten Hand. Diese Fragen entstammen einem standardisierten Fragebogen. Beziehen Sie sich bei der Beantwortung auf Ihr Befinden in der letzten Woche.

1. Wenn ich meine Aufmerksamkeit nicht auf die erkrankte Hand richte, liegt sie wie leblos neben mir.
   ☐ nie ☐ selten ☐ gelegentlich ☐ häufig ☐ fast immer ☐ immer
2. Die erkrankte Hand fühlt sich an als würde sie nicht mehr zu meinem restlichen Körper gehören.
   ☐ nie ☐ selten ☐ gelegentlich ☐ häufig ☐ fast immer ☐ immer
3. Ich muss meine ganze Aufmerksamkeit auf die erkrankte Hand richten, damit sie sich so bewegt, wie ich es will.
   ☐ nie ☐ selten ☐ gelegentlich ☐ häufig ☐ fast immer ☐ immer
4. Die erkrankte Hand bzw. der Arm macht Bewegungen die ich gar nicht machen wollte.
   ☐ nie ☐ selten ☐ gelegentlich ☐ häufig ☐ fast immer ☐ immer
5. Die erkrankte Hand fühlt sich wie abgestorben an.
   ☐ nie ☐ selten ☐ gelegentlich ☐ häufig ☐ fast immer ☐ immer
6. Ohne auf meine Hand zu gucken, kann ich nicht sagen, wo sie liegt.
   ☐ nie ☐ selten ☐ gelegentlich ☐ häufig ☐ fast immer ☐ immer

**6. Beim CRPS der unteren Extremität: Nun frage ich nach der Funktionsfähigkeit Ihres betroffenen Fußes. Die Fragen beziehen sich auf Ihr Befinden heute oder in der letzten Woche.
Bitte vergleichen Sie dabei Ihren Fuß mit dem Zustand vor der Erkrankung.**

6.1 Benutzen Sie Hilfsmittel für Ihren Fuß? *(Mehrfachnennung möglich)*

☐ ja → ☐ Rollstuhl
☐ Rollator
☐ eine Unterarmgehstütze
☐ zwei Unterarmgehstützen
☐ Orthesen
☐ Spezialschuhe

☐ nein

6.2 Wie gut können Sie mit Ihrem erkrankten Fuß auftreten?

☐ Kann ich gut.
☐ Kann ich eingeschränkt.
☐ Kann ich gar nicht.

Wenn eingeschränkt oder gar nicht möglich:
Was ist die Ursache hierfür? *(Mehrfachnennung möglich)*
☐ Schmerzen ☐ Kraft
☐ Beweglichkeit ☐ Keine Bewegungskontrolle möglich

Wenn Schmerzen vorhanden:
Wo sind die Schmerzen lokalisiert?
☐ Zehen ☐ Vorfuß ☐ Mittelfuß ☐Ferse ☐Sprunggelenk
☐ Außenkante

6.3 Können Sie ohne Beschwerden gehen?

☐ ja

☐ nein, ich kann nicht gehen
☐ aufgrund anderer Erkrankungen
☐ aufgrund des betroffenen Fußes

Wenn „nein“:

☐ > 1 km
☐ > 100 m
☐ Ich kann nur wenige Schritte ohne Beschwerden gehen.
☐ Ich kann *gar nicht* ohne Beschwerden gehen.

Was sind die Gründe hierfür? *(Mehrfachnennung möglich)*
☐ Schmerzen ☐ Kraft
☐ Beweglichkeit ☐ Keine Bewegungskontrolle möglich

6.4 Können Sie sich auf die Zehenspitzen stellen?

☐ ja → ☐ nur wenige Sekunden
→ ☐ länger als einige Sekunden

☐ nein, gar nicht.

Wenn nur wenige Sekunden oder gar nicht:
Was sind die Gründe hierfür? *(Mehrfachnennung möglich)*
☐ Schmerzen ☐ Kraft
☐ Beweglichkeit ☐ Keine Bewegungskontrolle möglich

6.5 Können Sie sich auf die Hacke stellen?

☐ ja → ☐ nur wenige Sekunden
→ ☐ länger als einige Sekunden

☐ nein, gar nicht.

Wenn nur kurz oder gar nicht:
Was sind die Gründe hierfür? *(Mehrfachnennung möglich)*
☐ Schmerzen ☐ Kraft
☐ Beweglichkeit ☐ Keine Bewegungskontrolle möglich

6.6 Wie stark sind Sie abschließend betrachtet bei der Benutzung Ihres erkrankten Fußes im Vergleich zum Zustand vor der Erkrankung beeinträchtigt?

☐ gar nicht beeinträchtigt
☐ wenig beeinträchtigt
☐ mittelmäßig beeinträchtigt
☐ stark beeinträchtigt?

6.7 Die folgenden Aussagen betreffen die Wahrnehmung des erkrankten Fußes. Bei manchen Patienten entsteht ein verändertes Gefühl des erkrankten Fußes. Diese Fragen entstammen einem standardisierten Fragebogen. Beziehen Sie sich bei der Beantwortung auf Ihr Befinden in der letzten Woche.

1. Wenn ich meine Aufmerksamkeit nicht auf den erkrankten Fuß richte, liegt er wie leblos neben mir.

   ☐ nie ☐ selten ☐ gelegentlich ☐ häufig ☐ fast immer ☐ immer

2. Der erkrankte Fuß fühlt sich an als würde er nicht mehr zu meinem restlichen Körper gehören.

   ☐ nie ☐ selten ☐ gelegentlich ☐ häufig ☐ fast immer ☐ immer

3. Ich muss meine ganze Aufmerksamkeit auf den erkrankten Fuß richten, damit er sich so bewegt wie ich es will.

   ☐ nie ☐ selten ☐ gelegentlich ☐ häufig ☐ fast immer ☐ immer

4. Der erkrankte Fuß macht Bewegungen, die ich gar nicht machen wollte.

   ☐ nie ☐ selten ☐ gelegentlich ☐ häufig ☐ fast immer ☐ immer

5. Der erkrankte Fuß fühlt sich wie abgestorben an.

   ☐ nie ☐ selten ☐ gelegentlich ☐ häufig ☐ fast immer ☐ immer

6. Ohne auf meinen Fuß zu gucken, kann ich nicht sagen, wo er liegt.

   ☐ nie ☐ selten ☐ gelegentlich ☐ häufig ☐ fast immer ☐ immer

Zuletzt würde Ich auf Ihre berufliche Situation und Arbeitsfähigkeit eingehen wollen.

7. Haben Sie vor Ihrer CRPS-Erkrankung gearbeitet?

☐ ja → ☐ vollschichtig
→ ☐ Teilzeit
☐ nein

Wenn nein:
Was war der Grund hierfür?

☐ Schule/Studium
☐ Ausbildung
☐ Rente
☐ Arbeitslos
☐ sonstige Gründe:

8. Frage nach körperlicher Beanspruchung an Arbeitsplätzen:

8.1 *Welche Aussage trifft auf Ihren Beruf zu, den Sie vor der CRPS-Erkrankung ausgeübt haben?*

☐ leichte Arbeit wie Handhaben leichter Werkstücke, Bedienen leichtgehender Steuerhebel oder ähnlicher mechanisch wirkender Einrichtungen, auch langandauerndes Stehen oder ständiges Umhergehen.

☐ Mittelschwere Arbeit wie Handhaben 1-3kg schwergehender Steuereinrichtungen, unbelastetes Gehen von Treppen und Leitern, Heben und Tragen von mittelschweren Lasten in der Ebene (von etwa 10-15 kg)

☐ Schwere Arbeiten wie Tragen von etwa 20-40 kg schweren Lasten in der Ebene oder Steigen unter mittleren Lasten und Handhaben von Werkzeugen (über 3 kg Gewicht) auch von Kraftwerkzeugen mit starker Rückstoßwirkung, Schaufeln, Graben, Hacken

☐ Schwerstarbeit wie Heben und Tragen von Lasten über 50 kg oder Steigen unter schwerer Last, vorwiegend Gebrauch schwerster Hämmer, schwerstes Ziehen und Schieben

8.2 *Wenn CRPS der oberen Extremität*:
Wie stark benötigten Sie Ihre Fingerfertigkeit in Ihrem Beruf?

☐ gar nicht ☐ wenig ☐ stark ☐ sehr stark belastet

9. Haben Sie heute bzw. nach dem CRPS wieder gearbeitet?

☐ nein

Wenn nein:
Was sind die Gründe hierfür?

☐ ich habe auch vorher nicht gearbeitet
☐ ich bin berentet, seit ____________________ (Datum)
☐ Altersrente
☐ vorzeitig berentet
☐ wegen CRPS
☐ aus anderen Gründen: ________________________

☐ ich bin arbeitsunfähig geschrieben
☐ AU wegen CRPS
☐ aus anderen Gründen

Seit wann?:
☐ Seit Beginn der Erkrankung/Seit dem Unfall.
☐ Erst seit ________________Monaten

Evtl. fragen, wie lange zwischendurch gearbeitet wurde:
____

☐ Ich bin nicht AU geschrieben, aber arbeitslos.
Intervall der AU erfragen: Vorher _______Monat AU gewesen
☐ Ich arbeite aus sonstigen Gründen nicht: _________________________

☐ ja
Seit wann arbeiten Sie wieder? *(Intervall der Arbeitsunfähigkeit in den erkrankten Jahren erfragen)*

Seit _______Jahren.

Wie lange waren sie arbeitsunfähig geschrieben:
_______Monate

☐ Ich habe die gleicher/ähnliche Arbeit wie früher.

☐ vollschichtig ☐ teilschichtig

☐ Ich habe eine andere Arbeit als früher.

☐ vollschichtig ☐ teilschichtig

☐ Ich habe eine Umschulung erhalten:
Umschulung von ____________________ zu ____________________
Beruf nach Umschulung ☐ vollschichtig ☐ teilschichtig

10. MDE/GDB

***Bei BG-Fall:* Haben Sie eine Minderung der Erwerbsfähigkeit?**

☐ ja
MdE: __%__ Seit:_______ Monaten
☐ nein
☐ aber beantragt Seit:_____ Monaten
☐ nicht beantragt

***Bei Nicht-BG-Fall:* Haben Sie einen Grad der Behinderung anerkannt bekommen?**

☐ ja
GdB-Grad: __ Seit:_______ Monaten
☐ nein
☐ aber beantragt Seit: ____ Monaten
☐ nicht beantragt

11. Sind Sie noch wegen der CRPS-Erkrankung in Behandlung?

☐ nein
☐ ja
☐ ja in der Bochumer Schmerzklinik
☐ ja, bei einem anderen Schmerztherapeuten
☐ ja, beim Hausarzt oder Facharzt

12. Haben Sie weitere der ff. Behandlungen (außer denen in Bochum) erfahren? Wenn ja, sagen Sie bitte, ob diese zu Verbesserungen geführt hat.

☐ nein
☐ ja

	nur ein- oder zweimal	häufiger	Kein Effekt	Etwas geholfen	Sehr geholfen
Injektionen					
Sympathikusblockaden, Stellatum, GLOA					
Plexus- oder Rückenmarkkatheter					
Spritzen an /in Gelenke					
Spritzen an Nerven					
Neurostimulation (SCS, PNS, andere)					
Operation wg. CRPS					
Amputation					
sonstige					

13. Sind Sie seit Beginn der Erkrankung noch einmal an dem betroffenen Arm/Bein operiert worden?

☐ nein
☐ ja
Wenn ja: **Wie oft?** ____Mal

Welche Eingriffe? ____________________________

Haben sich die CRPS-Beschwerden dadurch verändert?

☐ nein ☐ ja, verbessert ☐ ja, verschlimmert

Haben Sie noch Anmerkungen oder Wünsche?

____

____

____

Vielen Dank für Ihre Teilnahme! Alles Gute weiterhin!

Datum der Befragung: __________________

**Bei Kontrollpatienten wird die Erkrankung CRPS durch das entsprechende zu vergleichende Krankheitsbild (Neuralgie oder posttraumatische Gelenkschmerzen) ersetzt.*

Bergmannsheil
Berufsgenossenschaftliches Universitätsklinikum
UK RUB UNIVERSITÄTSKLINIKUM DER RUHR-UNIVERSITÄT BOCHUM

SCHMERZKLINIK RUHR
Bergmannsheil Berufsgenossenschaftliches Universitätsklinikum

Anamnese- und Untersuchungsbogen obere Extremität

Patientenaufkleber

Datum: ________________
Untersucher: ________________

Tagesfragebogen (heute) ausgefüllt?
□ ja
□ nein

1. Anamnese

Gewicht: ________________ kg
Größe: ________________ cm

Nebendiagnosen: ____
____
____

1.1. Erkrankte Seite: □ rechts □ links □ beidseits

1.2. Händigkeit: □ rechts □ links □ umgelernt

1.3. Auslösendes Ereignis / Primärer Auslöser (Mehrfachauswahl möglich):

□ **Fraktur, wenn ja wo?**<br>□ **keine**<br>□ **Operativ versorgt**	□ Clavicula<br>□ Scapula<br>□ Humerus<br>□ Unterarm→ □ Radius □ Ulna<br>□ MHK: ________________<br>□ Handwurzelknochen→ □ Skaphoid □ sonstige:________________<br>□ Phalanx ________________ → □ I □ II □ III □ IV □ V

1.4. Schmerzanamnese (seit wann bestehen die Schmerzen? ______________________)

**Lokalisation** →	□ Schulter	□ Oberarm	□ Unterarm	□ Hand	□ Finger/ Daumen
→	□ Oberflächlich	□ Tief			
**Schmerzqualität**	□ dumpf	□ brennend	□ stechend	□ pochend	□ ziehend
	□ drückend	□ klopfend	□ kribbelnd	□ schneidend	□ reißend
	□ krampfartig	□ Sonstige:	____________		
**Wann traten die heutigen Schmerzen das 1. Mal auf?**	____				

Schmerzklinik Ruhr. Dieser Bogen ist urheberrechtlich geschützt. Jeder Form der Vervielfältigung und Verbreitung sowie Übersetzung ist nicht erlaubt.

**Wie würden Sie Ihren Schmerz jetzt im Augenblick einschätzen?**	0	1	2	3	4	5	6	7	8	9	10
**Wie stark war der stärkste Schmerz in den letzten 4 Wochen?**	0	1	2	3	4	5	6	7	8	9	10
**Schmerzintensität (NRS)** **(im Mittel, letzen 4 Wochen)**	0	1	2	3	4	5	6	7	8	9	10

**Was <u>lindert</u> den Schmerz?**	□ Belastung/ Bewegung	□ körperlich<br>□ psychisch	□ Wärme<br>□ sonstiges	□ Kälte<br>_______	□ Ruhe	□ nichts
**Was <u>verstärkt</u> den Schmerz?**	□ Belastung/ Bewegung	□ körperlich<br>□ psychisch	□ Wärme<br>□ sonstiges	□ Kälte<br>_______	□ Ruhe	□ nichts

1.5. Medikamente aktuell: (Mehrfachauswahl möglich)

□ NSAID (inkl. Cox-2-Inhib.) □ TCA □ Metamizol
□ Opioide / WHO II □ SSRI □ Antikonvulsiva
□ Opioide / WHO III □ SNRI □ keine

2. <u>Budapestkriterien</u>

Patient → Symptome (Anamnese)

**Sensorik**				
□ seitengleiche normale Sensorik		□ Hyperalgesie	□ Hypästhesie	
□ Parästhesie		□ Dysästhesie	□ Allodynie	
**Vasomotorik**				
Hautfarbe	□ seitengleich	□ gerötet	□ livide	□ blass
Hauttemperatur	□ seitengleich	□ kälter	□ wärmer	
**Sudomotorik**				
Schweißsekretion	□ seitengleich	□ vermindert	□ vermehrt	
Ödem	□ nein	□ nur bei Belastung	□ in Ruhe	
**Trophik/Motorik**				
Tremor	□ nein	□ ja, →	□ beidseits	□ einseitig
Dystonie	□ nein	□ ja		
Kraftminderung	□ nein	□ ja, →	□ leicht	□ schwer
Nagelwuchs	□ seitengleich	□ vermindert	□ vermehrt	
Haarwuchs	□ seitengleich	□ vermindert	□ vermehrt	

Schmerzklinik Ruhr. Dieser Bogen ist urheberrechtlich geschützt. Jeder Form der Vervielfältigung und Verbreitung sowie Übersetzung ist nicht erlaubt.

Arzt → Zeichen (aktuell)

**Sensorik**				
□ seitengleiche normale Sensorik		□ Hyperalgesie	□Hypästhesie	
□ Parästhesie		□ Dysästhesie	□ Allodynie	
□ Druckschmerz über den Gelenken				
**Vasomotorik**				
Hautfarbe	□ seitengleich	□ gerötet	□ livide	□ blass
Hauttemperatur	□ seitengleich	□ kälter	□ wärmer	
**Sudomotorik**				
Schweißsekretion	□ seitengleich	□ vermindert	□ vermehrt	
Ödem	□ nein	□ nur bei Belastung	□ in Ruhe	
**Trophik/Motorik**				
Tremor	□ nein	□ ja, →	□ beidseits	□ einseitig
Dystonie	□ nein	□ ja		
Kraftminderung	□ nein	□ ja, →	□ leicht	□ schwer
Nagelwuchs	□ seitengleich	□ vermindert	□ vermehrt	
Haarwuchs	□ seitengleich	□ vermindert	□ vermehrt	
**Gebrauchsspuren/ Beschwielung**	□ Seitengleich	□ Vermehrt	□ Vermindert	
**Atrophie**	□ thenar	□ hypothenar	□ Mm. Interosseii	
	□ sonst. Areale: ____________________			

3. klinischer Befund der oberen Extremität

Schulter		**Anteversion/ Retroversion**	**Abduktion/ Adduktion**	**IRO/ARO (adduzierter Arm, Ellenbogen in 90° Flexion)**
	Rechts	__ / __ / __	__ / __ / __	__ / __ / __
	Links	__ / __ / __	__ / __ / __	__ / __ / __
	Schürzengriff<br>□ möglich<br>□ nicht möglich		Nackengriff<br>□ möglich<br>□ nicht möglich	

Ellenbogen		**Extension/ Flexion**	**Pronation/ Supination**
	Rechts	__ / __ / __	__ / __ / __
	Links	__ / __ / __	__ / __ / __
Hand		**Dorsalextension/ Palmarflexion**	**Radialabduktion/Ulnarabduktion**
	Rechts	__ / __ / __	__ / __ / __
	Links	__ / __ / __	__ / __ / __

Schmerzklinik Ruhr. Dieser Bogen ist urheberrechtlich geschützt. Jeder Form der Vervielfältigung und Verbreitung sowie Übersetzung ist nicht erlaubt.

Finger-Hohlhand-Abstand : ____
(Abstand zwischen Fingerspitze und Handflächenmitte palmar)

Faustschluss: □ möglich □ nicht möglich

Finger

Daumenopposition
(bitte ankreuzen, welche Langfingerkuppen mit der Daumenspitze auf <u>betroffener Seite</u> erreicht werden können)

	Dig.V	Dig. IV	Dig. III	Dig. II	□ nicht möglich
**Betroffene Seite**	□ ja	□ ja	□ ja	□ ja	

Betroffene Seite	MCP	PIP	DIP
**Dig. I**	__ /__ /__	__ /__ /__ **(IP)**	
**Dig. II**	__ /__ /__	__ /__ /__	__ /__ /__
**Dig. III**	__ /__ /__	__ /__ /__	__ /__ /__
**Dig. IV**	__ /__ /__	__ /__ /__	__ /__ /__
**Dig. V**	__ /__ /__	__ /__ /__	__ /__ /__

<u>Umfänge der oberen Extremität (**hängender Arm**)</u>

<u>Umfang (cm)</u>	<u>Ipsilateral</u>	<u>Kontralateral</u>
**Oberarm** (15cm proximal der Ellenbeuge)		
**Ellenbogengelenk**		
**Unterarm** (10cm distal der Ellenbeuge)		
**Handgelenk**		
**Mittelhand** ohne Daumen		
**Dig. II** (mm)		
**Dig. IV** (mm)		

Druck-schmerz

Besteht eine Druckdolenz bei leichtem Druck im Seitenvergleich?
*(druckschmerzhafte **<u>Gelenke</u>** im Bild mit einem X markieren)*

Rechts

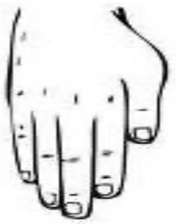

Links

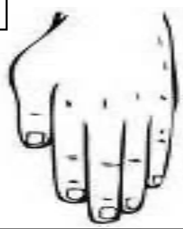

Schmerzklinik Ruhr. Dieser Bogen ist urheberrechtlich geschützt. Jeder Form der Vervielfältigung und Verbreitung sowie Übersetzung ist nicht erlaubt.

Bergmannsheil
Berufsgenossenschaftliches Universitätsklinikum
UK RUB UNIVERSITÄTSKLINIKUM DER RUHR-UNIVERSITÄT BOCHUM

SCHMERZKLINIK RUHR
Bergmannsheil

□ **Allodynie** XXX : □ **radikulär:** _________ □ **Nervengebiet**
(Bitte einzeichnen!) **(Segment)** ________________

□ **Hypästhesie** ///: □ **radikulär:** _________ □ **Nervengebiet**
(Bitte einzeichnen!) **(Segment)** ________________

□ **Dysästhesie** >>>: □ **radikulär:** _________ □ **Nervengebiet**
(Bitte einzeichnen!) **(Segment)** ________________

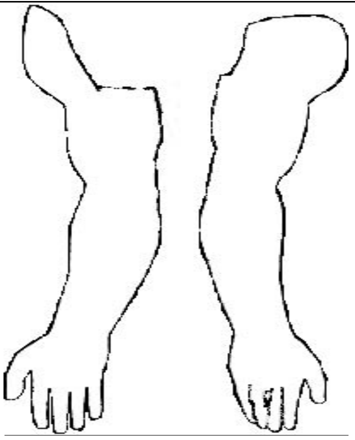

Hoffmann- Tinel Zeichen: □ neg □ pos **Phalentest:** □ neg □ pos
→ □ N. axillaris →□ N. radialis
→ □ N. medianus →□ N. ulnaris

Reflexe:

Bicepssehnenreflex:

□ seitengleich
□ vermindert (rechts/ links/ bds.)
□ aufgehoben (rechts/ links/ bds.)

Pallästhesie:

Processus styloideus radii____/8

4. Verdachtsdiagnose/ Arbeitshypothese

Schmerzklinik Ruhr. Dieser Bogen ist urheberrechtlich geschützt. Jeder Form der Vervielfältigung und Verbreitung sowie Übersetzung ist nicht erlaubt.

Weitere Untersuchungen:

	angemeldet	Befund
**Röntgen**	☐	
**Szintigraphie**	☐	
**MRT**	☐	
**QST**	☐	
**Elektroneurographie**	☐	
**Weitere Untersuchung:**	☐	
	☐	

Klinik für Anästhesiologie,
Intensiv-, Palliativ- und Schmerzmedizin

Direktor:
Prof. Dr. med. Peter Zahn

Abteilung für Schmerzmedizin

Leitender Arzt:
Prof. Dr. med. Christoph Maier
Telefon:0234 / 302-6366
Fax:0234 / 302-6367
E-Mail:christoph.maier@ruhr-uni-bochum.de

Schmerzklinik Ruhr. Dieser Bogen ist urheberrechtlich geschützt. Jeder Form der Vervielfältigung und Verbreitung sowie Übersetzung ist nicht erlaubt.

Anamnese- und Untersuchungsbogen untere Extremität

Patientenaufkleber

Datum: ________________
Untersucher: ________________

Tagesfragebogen (heute) ausgefüllt? □ ja
□ nein

Anamnese

Gewicht: _______________ kg **Größe:** _______________ cm

Nebendiagnosen: ____

____

____

Erkrankte Seite: □ rechts □ links □ beidseits

Erkrankungsdauer: _______ Monate Unfalldatum:______________

Spielbein: □ rechts □ links □ umgelernt

Ereignis:

**Auslösendes Ereignis**			
□ Distorsionstrauma		□ Weichteiltrauma (z.B. Tendovaginitis)	□sonstige_____________
**Fraktur, wenn ja Lokalisation** ____			
□ einfach	□ mehrfach	□ Trümmerfraktur	□ mit Gelenkbeteiligung
Versorgung:		□ operativ	□ konservativ
**Sonstiges auslösendes Ereignis(z.B.Apoplex):**______________________________________			
□ medikamenteninduziert		□ kein Trauma erinnerlich	

**Schuhversorgung**		
□ keine besondere Versorgung	□ Einlagen	□ orthopädisches Schuhwerk
□ sonstiges_________		
**Fußbelastung**		
□ Vollbelastung	□ Teilbelastung ____kg	□ keine Belastung

Schmerzanamnese

**Fühlen Sie den Schmerz eher in der Tiefe oder oberflächlich?**				
□ Tiefe	□ oberflächlich	□ beides		
**Trat der Schmerz direkt nach dem Trauma/OP auf oder mit zeitlicher Verzögerung?**				
□ direkt	□ nach_______Wochen			
**Lokalisation**				
□ Hüfte	□ Oberschenkel	□ Knie	□ Unterschenkel	□ Fuß
□ OSG	□ USG	□ Vorfuß	□ Mittelfuß	□ Ferse

Schmerzklinik Ruhr. Dieser Bogen ist urheberrechtlich geschützt. Jeder Form der Vervielfältigung und Verbreitung sowie Übersetzung ist nicht erlaubt.

**Schmerzqualität**	□ dumpf<br>□ drückend<br>□ krampfartig	□ brennend<br>□ klopfend<br>□ Sonstige:	□ stechend<br>□ kribbelnd<br>____________	□ pochend<br>□ schneidend	□ ziehend<br>□ reißend
**Wann traten die heutigen Schmerzen das 1. Mal auf?**					

**Wie würden Sie Ihren Schmerz jetzt im Augenblick einschätzen?**	0	1	2	3	4	5	6	7	8	9	10
**Wie stark war der stärkste Schmerz in den letzten 4 Wochen?**	0	1	2	3	4	5	6	7	8	9	10
**Schmerzintensität (NRS) (im Mittel, letzen 4 Wochen)**	0	1	2	3	4	5	6	7	8	9	10

**Was <u>lindert</u> den Schmerz?**	□ Belastung/	□ körperlich<br>□ psychisch	□ Wärme<br>□ sonstiges	□ Kälte<br>_______	□ Ruhe	□ nichts
**Was <u>verstärkt</u> den Schmerz?**	□ Belastung/ Bewegung	□ körperlich<br>□ psychisch	□ Wärme<br>□ sonstiges	□ Kälte<br>_______	□ Ruhe	□ nichts

<u>Medikamente aktuell:</u> (Mehrfachauswahl möglich)

☐ NSAID (inkl. Cox-2-Inhib.)	☐ TCA	☐ Metamizol
☐ Opioide / WHO II	☐ SSRI	☐ Antikonvulsiva
☐ Opioide / WHO III	☐ SNRI	☐ keine

<u>Budapestkriterien</u>
Patient → Symptome (Anamnese):

**Sensorik**					
□ seitengleiche normale Sensorik		□ Hyperalgesie	□ Hypästhesie		
□ Parästhesie		□ Dysästhesie	□ Allodynie		
**Vasomotorik**					
Hautfarbe	□ seitengleich	□ gerötet	□ livide		□ blass
Hauttemperatur	□ seitengleich	□ kälter	□ wärmer		
**Sudomotorik**					
Schweißsekretion	□ seitengleich	□ vermindert	□ vermehrt		
Ödem	□ nein	□ nur bei Belastung	□ in Ruhe		
**Trophik/Motorik**					
Tremor	□ nein	□ ja, →	□ beidseits	□ einseitig	
Dystonie	□ nein	□ ja			
Kraftminderung	□ nein	□ ja, →	□ leicht	□ schwer	
Nagelwuchs	□ seitengleich	□ vermindert	□ vermehrt		
Haarwuchs	□ seitengleich	□ vermindert	□ vermehrt		

Schmerzklinik Ruhr. Dieser Bogen ist urheberrechtlich geschützt. Jeder Form der Vervielfältigung und Verbreitung sowie Übersetzung ist nicht erlaubt.

Arzt → Zeichen (aktuell):

**Sensorik**						
□ seitengleiche normale Sensorik		□ Hyperalgesie		□ Hypästhesie		
□ Parästhesie		□ Dysästhesie		□ Allodynie		
□ Druckschmerz über den Gelenken						
**Vasomotorik**						
Hautfarbe		□ seitengleich		□ gerötet	□ livide	□ blass
Hauttemperatur		□ seitengleich		□ kälter	□ wärmer	
**Sudomotorik**						
Schweißsekretion	□ seitengleich	□ vermindert		□ vermehrt		
Ödem	□ nein	□ nur bei Belastung		□ in Ruhe		
**Trophik/Motorik**						
Tremor	□ nein	□ ja, →	□ beidseits		□ einseitig	
Dystonie	□ nein	□ ja				
Kraftminderung	□ nein	□ ja, →	□ leicht		□ schwer	
Nagelwuchs	□ seitengleich	□ vermindert		□ vermehrt		
Haarwuchs	□ seitengleich	□ vermindert		□ vermehrt		

Klinischer Befund:

**Deformitäten**						
Fuß	□ hallux valgus<br>□ Zehendeformität	□ hallux rigidus<br>□ keine	□ Hohlfuß	□ Senkfuß	□ Spreizfuß	□ Knickfuß
Knie	□ genu varus	□ rechts	□ links	□ bds		
	□ genu valgus	□ rechts	□ links	□ bds		
Hüfte	□ coxae varae	□ rechts	□ links	□ bds		
	□ coxae valgae	□ rechts	□ links	□ bds		
**Fußgewölbe**						
lateral	□ intakt	□ atrophiert				
medial	□ intakt	□ atrophiert				

Hüftgelenke:

	Rechts			Links		
Extension/ Flexion (Abb. 1a u. 1b)						
Abduktion/ Adduktion (Abb. 2)						
Außenrotation/ Innenrotation						

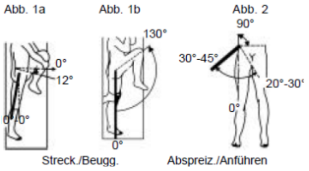

Kniegelenk:

Extension/ Flexion (Abb. 1a u. 1b)						

Obere Sprunggelenk:

Heben/ Senken						

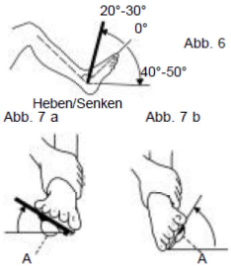

Untere Sprunggelenke:

Ges.-Beweglichkeit (Fußaußenrot.)		

Zehengelenke:

Beweglichkeit	□ o.p.B.	□ o.p.B.

Schmerzklinik Ruhr. Dieser Bogen ist urheberrechtlich geschützt. Jeder Form der Vervielfältigung und Verbreitung sowie Übersetzung ist nicht erlaubt.

Umfang in cm:

20cm ob. inn. Kniegelenkspalt		
10cm ob. inn. Kniegelenkspalt		
Kniescheibenmitte		
15cm unterh. inn. Kniegelenkspalt		
Unterschenkel, kleinster Umfang		
Knöchel, Rist über Kahnbeinl		
Vorfußballen		

Fußpulse: A.dorsalis pedis
- → ☐ bds palpabel
- → ☐ nur ipsilateral palpabel
- → ☐ nur kontralateral palpabel
- → Lagerungsprobe nach Ratschow: ☐____

A.tibialis posterior
- → ☐ bds palpabel
- → ☐ nur ipsilateral palpabel
- → ☐ nur kontralateral palpabel

Beschwielung: ☐normal im Seitenvergleich ☐vermehrt, wo?___________ ☐vermindert________

Achsabweichung: ☐nein ☐fibular ___° ☐tibial ___°

Gangvariationen: ☐Zehenspitzengang ☐__________ ▫ o.p.B. ☐Fersengang ☐__________ ☐o.p.B.

Standvariation: ☐ Einbeinstand ☐__________☐ o.p.B. ☐Hocke ☐______________ ☐o.p.B.

Abrollbewegung: ☐nicht möglich__________ ☐o.p.B.

Sensorik:

Bitte einzeichnen: XXX Allodynie /// Dysästhesie \\\ Hypästhesie

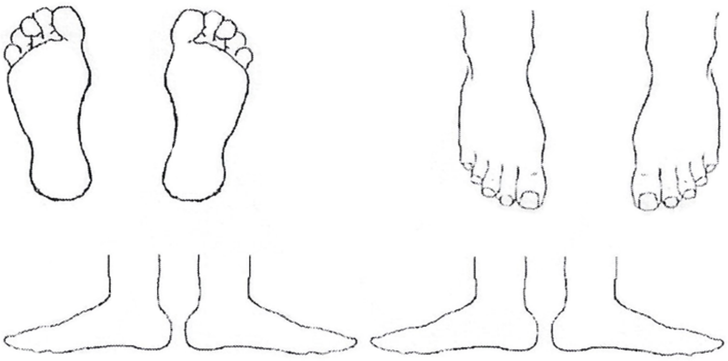

Ansicht von tibial Ansicht von fibular

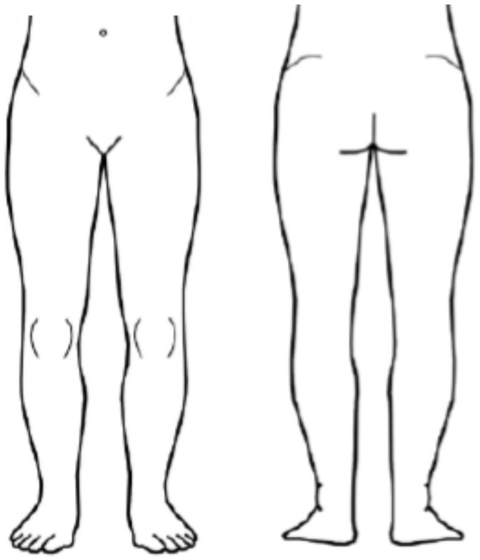

Reflexe:

**Patellarsehnenreflex**				
	☐seitengleich			
	☐vermindert	☐bds.	☐rechts	☐links
**Achillessehnenreflex**				
	☐seitengleich			
	☐vermindert	☐bds.	☐rechts	☐links
**Babinski:**	☐ neg	☐ pos		

Schmerzklinik Ruhr. Dieser Bogen ist urheberrechtlich geschützt. Jeder Form der Vervielfältigung und Verbreitung sowie Übersetzung ist nicht erlaubt.

Pallästhesie:

Malleolus medialis links ____/8 Malleolus medialis rechts ____/8

Funktionsprüfung:

Trendelenburg	□ neg	□ pos
Thomas Handgriff (bei Hyperlordosierung)	□ neg	□ pos
Laségue	□ neg	□ pos
Payr-Zeichen	□ neg	□ pos
Steinmann I	□ neg	□ pos
Steinmann II	□ neg	□ pos
Turner: (Valgus- /Varus-Test)	□ neg	□ pos
Lachmann: (Schublade bei 30°)	□ neg	□ pos
Pivot-Shift	□ neg	□ pos
Böhler-Test	□ neg	□ pos

Kraftprüfung:

L1 (Hüftbeugung)	☐unauffällig	Kraftgrad ____/5
L2 (Adduktoren)	☐unauffällig	Kraftgrad ____/5
L3 (Kniestreckung)	☐unauffällig	Kraftgrad ____/5
L4 (Dorsalextension Fuß)	☐unauffällig	Kraftgrad ____/5
L5 (Extension Großzehe)	☐unauffällig	Kraftgrad ____/5
S1 (Plantarflexion Fuß)	☐unauffällig	Kraftgrad ____/5

Druckschmerzschwellen in kPA:

Rechts			
MTP I			
MTP II			
MTP III			
MTP IV			
MTP V			
M. adductor hallucis			
Calcaneus			

Links			
MTP I			
MTP II			
MTP III			
MTP IV			
MTP V			
M. adductor hallucis			
Calcaneus			

Schmerzhaftigkeit nach PPT (NRS, 0-10):

links _____ rechts: _____

<u>Verdachtsdiagnose/ Arbeitshypothese</u>

	angemeldet	Befund
**Röntgen**	□	
**Szintigraphie**	□	
**MRT**	□	
**QST**	□	
**Elektroneurographie**	□	
**Weitere Untersuchung:**	□	

Schmerzklinik Ruhr. Dieser Bogen ist urheberrechtlich geschützt. Jeder Form der Vervielfältigung und Verbreitung sowie Übersetzung ist nicht erlaubt.

Mit den folgenden Fragen, möchten wir von Ihnen erfahren, welche Alltagstätigkeiten Sie mit beiden Händen in der vergangenen Woche trotz Ihrer Erkrankung ausführen konnten. Bitte geben Sie für die linke und rechte Hand getrennt an, wie stark Sie bei der Durchführung der einzelnen Tätigkeiten beeinträchtigt sind. Falls eine der Tätigkeiten (z.B. Computertastatur bedienen) für Sie keine Bedeutung hat, streichen Sie diese Tätigkeit bitte deutlich erkennbar durch.

mit der **linken** Hand: ohne Probleme durchführbar					z.Z. gar nicht durchführbar		mit der **rechten** Hand: ohne Probleme durchführbar					z.Z. gar nicht durchführbar
○	○	○	○	○	○	Flasche mit Drehverschluss öffnen	○	○	○	○	○	○
○	○	○	○	○	○	Zeitung oder Buch halten	○	○	○	○	○	○
○	○	○	○	○	○	Computer-Tastatur bedienen	○	○	○	○	○	○
○	○	○	○	○	○	Tür aufschließen	○	○	○	○	○	○
○	○	○	○	○	○	schwere Tür aufdrücken	○	○	○	○	○	○
○	○	○	○	○	○	gefüllten Suppenteller tragen	○	○	○	○	○	○
○	○	○	○	○	○	leichte Hausarbeit verrichten (z.B. abtrocknen, Staub wischen)	○	○	○	○	○	○
○	○	○	○	○	○	schwere Hausarbeit verrichten (z.B. Betten beziehen, Fenster putzen)	○	○	○	○	○	○
○	○	○	○	○	○	Einkaufstaschen tragen	○	○	○	○	○	○
○	○	○	○	○	○	volle Getränkekiste anheben	○	○	○	○	○	○
○	○	○	○	○	○	Glühbirne auswechseln	○	○	○	○	○	○
○	○	○	○	○	○	Haare fönen oder frisieren	○	○	○	○	○	○
○	○	○	○	○	○	Hemd oder eine Bluse zuknöpfen	○	○	○	○	○	○
○	○	○	○	○	○	mit Messer oder Schere schneiden	○	○	○	○	○	○
○	○	○	○	○	○	Auto oder Fahrrad lenken	○	○	○	○	○	○
○	○	○	○	○	○	Schuhe zubinden	○	○	○	○	○	○

Mit den folgenden Fragen, möchten wir von Ihnen erfahren, welche Alltagstätigkeiten Sie mit beiden Füssen in der vergangenen Woche trotz Ihrer Erkrankung ausführen konnten. Bitte geben Sie für den linken und rechten Fuß getrennt an, wie stark Sie bei der Durchführung der einzelnen Tätigkeiten beeinträchtigt sind. Falls eine der Tätigkeiten (z.B. Auto- oder Fahrradpedal benutzen) für Sie keine Bedeutung hat, streichen Sie diese Tätigkeit bitte deutlich erkennbar durch.

mit dem **linken** Fuß							mit dem **rechten** Fuß					
ohne Probleme durchführbar					z.Z. gar nicht durchführbar		ohne Probleme durchführbar					z.Z. gar nicht durchführbar
○	○	○	○	○	○	beim Sitzen den Fuß aufsetzen	○	○	○	○	○	○
○	○	○	○	○	○	einen Strumpf anziehen	○	○	○	○	○	○
○	○	○	○	○	○	einen festen Straßenschuh tragen	○	○	○	○	○	○
○	○	○	○	○	○	auf einem Bein stehen	○	○	○	○	○	○
○	○	○	○	○	○	auf Zehenspitzen stehen	○	○	○	○	○	○
○	○	○	○	○	○	ein Auto- oder Fahrradpedal benutzen	○	○	○	○	○	○
○	○	○	○	○	○	über kleine Erhebungen gehen (z.B. Türschwellen, Teppichkanten)	○	○	○	○	○	○
○	○	○	○	○	○	einen Gegenstand zur Seite treten (z.B. Dosen, Bälle, Zweige)	○	○	○	○	○	○
○	○	○	○	○	○	beim Sitzen mit dem Fuß wippen	○	○	○	○	○	○

zehn Treppenstufen herauf steigen

○ ja, problemlos
○ ja, ...
○ nur mit Hilfsmittel (z.B. Gehstützen)
○ nein

zehn Treppenstufen herab steigen

○ ja
○ ja, ...
○ nur mit Hilfsmittel (z.B. Gehstützen)
○ nein

Mit den folgenden Fragen, möchten wir von Ihnen erfahren, welche Alltagstätigkeiten Sie mit beiden Füssen in der vergangenen Woche trotz Ihrer Erkrankung ausführen konnten. Bitte geben Sie für den linken und rechten Fuß getrennt an, wie stark Sie bei der Durchführung der einzelnen Tätigkeiten beeinträchtigt sind. Falls eine der Tätigkeiten (z.B. Auto- oder Fahrradpedal benutzen) für Sie keine Bedeutung hat, streichen Sie diese bitte deutlich erkennbar durch.

mit dem **linken** Fuß							mit dem **rechten** Fuß					
ohne Probleme durchführbar					z.Z. gar nicht durchführbar		ohne Probleme durchführbar					z.Z. gar nicht durchführbar
○	○	○	○	○	○	beim Sitzen den Fuß aufsetzen	○	○	○	○	○	○
○	○	○	○	○	○	einen Strumpf anziehen	○	○	○	○	○	○
○	○	○	○	○	○	einen festen Straßenschuh tragen	○	○	○	○	○	○
○	○	○	○	○	○	auf einem Bein stehen	○	○	○	○	○	○
○	○	○	○	○	○	auf Zehenspitzen stehen	○	○	○	○	○	○
○	○	○	○	○	○	ein Auto- oder Fahrradpedal benutzen	○	○	○	○	○	○
○	○	○	○	○	○	über kleine Erhebungen gehen (z.B. Türschwellen, Teppichkanten)	○	○	○	○	○	○
○	○	○	○	○	○	einen Gegenstand zur Seite treten (z.B. Dosen, Bälle, Zweige)	○	○	○	○	○	○
○	○	○	○	○	○	beim Sitzen mit dem Fuß wippen	○	○	○	○	○	○

Können Sie zehn Treppenstufen herauf steigen?

- ○ ja, problemlos
- ○ ja, aber mit Problemen (z.B. ziehe ich ein Bein jede Stufe einzeln hoch)
- ○ ja, aber nur mit Hilfsmittel (z.B. Gehstützen)
- ○ nein, allein gar nicht

Können Sie zehn Treppenstufen herab steigen?

- ○ ja, problemlos
- ○ ja, aber mit Problemen (z.B. ziehe ich ein Bein nach jeder Stufe einzeln nach)
- ○ ja, aber nur mit Hilfsmittel (z.B. Gehstützen)
- ○ nein, allein gar nicht

Können Sie auf eine Leiter steigen?

- ○ Ja
- ○ ja, aber mit Problemen (z.B. ziehe ich ein Bein nach)
- ○ nein

Brauchen Sie für das Gehen in Ihrer Wohnung ein Hilfsmittel?

○ nein ○ ja Wenn ja, welches:

- ○ Eine Unterarmgehstütze
- ○ Zwei Unterarmgehstützen
- ○ Orthese (z.B. äußere Knieschiene
- ○ Rollator
- ○ Rollstuhl
- ○ Sonstiges: ______________________________

Brauchen Sie für alltägliche Besorgungen, die Sie außer Haus zu Fuß erledigen, ein Hilfsmittel?

○ nein ○ ja Wenn ja, welches:

- ○ Eine Unterarmgehstütze
- ○ Zwei Unterarmgehstützen
- ○ Orthese (z.B. äußere Knieschiene
- ○ Rollator
- ○ Rollstuhl
- ○ Sonstiges: ______________________________
- ○ Ich verlasse meine Wohnung nicht

© Frettlöh, J. & Maier, C. 2003; Version 4

ERSTERFASSUNG

Disabilities of the
Arm, Shoulder and Hand Instrument

May 1997 Version 2.0

Einschätzung des gegenwärtigen Gesundheitszustandes S. 2 - 9
Disabilities of the Arm, Shoulder and Hand Modul S. 10 - 14
Angaben zur Person S. 15 - 16

D A S H - Fragebogen

(Deutsche Version GERMANN, HARTH, WIND, DEMIR)

Juni 2001 Deutsche-Version 2.0

Copyright 1997

American Academy of Orthopaedic Surgeons
Institute for Work and Health, Toronto
American Association for Hand Surgery
American Society for Surgery of the Hand
American Orthopaedic Society for Sports Medicine
American Shoulder and Elbow Surgeons
Arthroscopy Association of North America
American Society of Plastic and Reconstructive Surgeons

Klinik für Hand-, Plastische und Rekonstruktive Chirurgie
– Schwerbrandverletztenzentrum – BG-Unfallklinik Ludwigshafen
Klinik für Plastische und Handchirurgie der Universität Heidelberg

Ludwig-Guttmann-Strasse 13
67071 Ludwigshafen

Dieser Fragebogen enthält die
SF-36 Standard German Version 1.0
in den Fragen 15 bis 25. Vervielfältigung mit Erlaubnis
des Medical Outcomes Trust (Copyright 1992)

Alle Rechte vorbehalten

Erfassung des gegenwärtigen Gesundheitszustands

**ID Nr.:**	**BG:**
	**Kasse:**
**Diagnose:**	
	**Alter:**
**ICD – Codierung:**	**Datum:**

Wir bitten Sie, diesen Fragebogen auszufüllen, so dass wir Ihren allgemeinen Gesundheitszustand und Ihr aktuelles gesundheitliches Problem besser erfassen, verstehen und behandeln können.
Das Ausfüllen dieses Bogens ist freiwillig. Ihre Antworten werden natürlich streng vertraulich behandelt.
Das Ausfüllen dauert etwa 15-20 Minuten.

Beantworten Sie bitte jede Frage. Einige Fragen scheinen sich zu ähneln, aber in der Tat ist jede anders.

Bitte geben Sie Ihre Antworten, indem Sie das passende Kästchen bzw. die passende Ziffer markieren.

Es gibt keine richtigen oder falschen Antworten. Falls Sie nicht sicher sind, wie Sie eine Frage beantworten sollen, markieren Sie die Antwort, welche am ehesten für Sie zutrifft und schreiben Sie Ihre Kommentare dazu, auch diese werden berücksichtigt.

Ihre **rechte Körperseite** betreffend – markieren Sie bitte die Körperregionen, welche Sie bei ihren Aktivitäten behindern oder einschränken

Ihre **linke Körperseite** betreffend – markieren Sie bitte die Körperregionen, welche Sie bei ihren Aktivitäten behindern oder einschränken

Rechts		Links
Hals ☐		☐ Hals
Schulterbereich ☐		☐ Schulterbereich
Ellenbogen / Unterarm ☐		☐ Ellenbogen / Unterarm
Handgelenk / Hand ☐		☐ Handgelenk / Hand
Hüfte ☐		☐ Hüfte
Oberschenkel ☐		☐ Oberschenkel
Kniebereich ☐		☐ Kniebereich
Unterschenkel ☐		☐ Unterschenkel
Sprunggelenk / Fußbereich ☐		☐ Sprunggelenk / Fußbereich

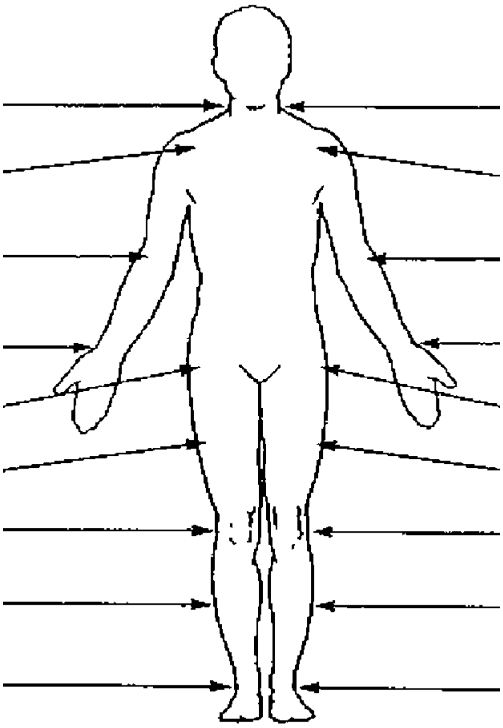

Ihren **Rücken** betreffend – markieren Sie bitte die Körperregionen, welche Sie bei ihren Aktivitäten behindern oder einschränken

- Hals ☐
- Oberer Rückenbereich ☐
- Unterer Rückenbereich ☐

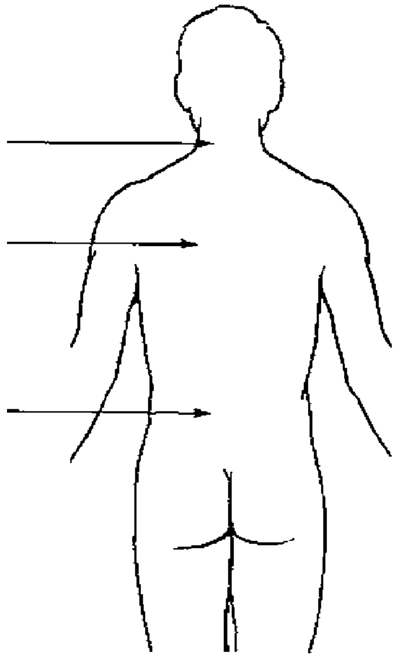

Ihre **Hände** betreffend – markieren Sie bitte die Regionen, mit den stärksten Schmerzen

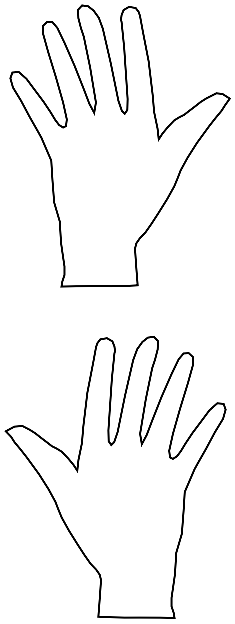

In den Fragen 1 bis 14 sind einige häufig auftretende Erkrankungen aufgelistet.

Bitte geben Sie in der ersten Spalte mit „ja“ oder „nein“ an, ob die jeweilige Erkrankung oder Beschwerde auf Sie zutrifft. Ist dies der Fall, dann markieren Sie bitte in der zweiten Spalte, ob Sie deswegen Medikamente einnehmen oder in Behandlung sind. In der dritten Spalte beantworten Sie bitte, ob Sie durch diese Gesundheitsstörung in Ihren Alltagstätigkeiten eingeschränkt sind.

	**Trifft dies auf Sie zu?**		**Sind Sie deswegen in Behandlung?**		**Werden Sie dadurch in Ihren Aktivitäten eingeschränkt?**	
**1. Herzerkrankung**	ja	nein	ja	nein	ja	nein
**2. Hoher Blutdruck**	ja	nein	ja	nein	ja	nein
**3. Atemwegserkrankung**	ja	nein	ja	nein	ja	nein
**4. Diabetes**	ja	nein	ja	nein	ja	nein
**5. Magengeschwür**	ja	nein	ja	nein	ja	nein
**6. Nierenerkrankung**	ja	nein	ja	nein	ja	nein
**7. Lebererkrankung**	ja	nein	ja	nein	ja	nein
**8. Bluterkrankungen**	ja	nein	ja	nein	ja	nein
**9. Krebs**	ja	nein	ja	nein	ja	nein
**10. Depression**	ja	nein	ja	nein	ja	nein
**11. Arthrose**	ja	nein	ja	nein	ja	nein
**12. Rückenschmerzen**	ja	nein	ja	nein	ja	nein
**13. Rheumatoide Arthritis**	ja	nein	ja	nein	ja	nein
**14. Andere Gesundheitsstörungen** (Bitte genauer angeben)						
....	ja	nein	ja	nein	ja	nein
....	ja	nein	ja	nein	ja	nein

15. Wie würden Sie Ihren Gesundheitszustand im Allgemeinen beschreiben?
(Bitte kreuzen Sie nur ein Kästchen an)

Ausgezeichnet	Sehr gut	Gut	Weniger gut	Schlecht
☐	☐	☐	☐	☐

16. Im Vergleich zum vergangenen Jahr, wie würden Sie Ihren derzeitigen Gesundheitszustand beschreiben?
(Bitte kreuzen Sie nur ein Kästchen an)

Derzeit viel besser als vor einem Jahr	☐
Derzeit etwas besser als vor einem Jahr	☐
Etwa so wie vor einem Jahr	☐
Derzeit etwas schlechter als vor einem Jahr	☐
Derzeit viel schlechter als vor einem Jahr	☐

17. Im folgenden sind einige Tätigkeiten beschrieben, die Sie vielleicht an einem normalen Tag ausüben. Sind Sie durch Ihren derzeitigen Gesundheitszustand bei diesen Tätigkeiten eingeschränkt? Wenn ja, wie stark?
(Bitte kreuzen Sie in jeder Zeile nur ein Kästchen an)

	**Ja, stark einge-schränkt**	**Ja, etwas einge-schränkt**	**Nein, überhaupt nicht einge-schränkt**
a. **Anstrengende Tätigkeiten,** z.B. schnell laufen, schwere Gegenstände heben, anstrengenden Sport treiben	☐	☐	☐
b. **Mittelschwere Tätigkeiten,** z.B. einen Tisch verschieben, staubsaugen, kegeln, Golf spielen	☐	☐	☐
c. Einkaufstaschen heben oder tragen	☐	☐	☐
d. **Mehrere** Treppenabsätze steigen	☐	☐	☐
e. **Einen** Treppenabsatz steigen	☐	☐	☐
f. Sich beugen, knien, bücken	☐	☐	☐
g. **Mehr als 1 Kilometer** zu Fuß gehen	☐	☐	☐
h. **Mehrere Straßenkreuzungen** weit zu Fuß gehen	☐	☐	☐
i. **Eine Straßenkreuzung** weit zu Fuß gehen	☐	☐	☐
j. Sich baden oder anziehen	☐	☐	☐

18. Hatten Sie in den vergangenen 4 Wochen aufgrund Ihrer körperlichen Gesundheit irgendwelche Schwierigkeiten bei der Arbeit oder anderen alltäglichen Tätigkeiten im Beruf bzw. zu Hause?

(Bitte kreuzen Sie in jeder Zeile nur ein Kästchen an)

SCHWIERIGKEITEN	Ja	Nein
Ich konnte nicht **so lange** wie üblich tätig sein	☐	☐
Ich habe **weniger geschafft** als ich wollte	☐	☐
Ich konnte **nur bestimmte** Dinge tun	☐	☐
Ich hatte **Schwierigkeiten** bei der Ausführung (z. B. musste ich mich besonders anstrengen)	☐	☐

19. Hatten Sie in den vergangenen 4 Wochen aufgrund seelischer Probleme irgendwelche Schwierigkeiten bei der Arbeit oder anderen alltäglichen Tätigkeiten im Beruf bzw. zu Hause (z. B., weil Sie sich niedergeschlagen oder ängstlich fühlten)?

(Bitte kreuzen Sie in jeder Zeile nur ein Kästchen an)

SCHWIERIGKEITEN	Ja	Nein
Ich konnte nicht **so lange** wie üblich tätig sein	☐	☐
Ich habe **weniger geschafft** als ich wollte	☐	☐
Ich konnte nicht so **sorgfältig** wie üblich arbeiten	☐	☐

20. Wie sehr haben Ihre körperliche Gesundheit oder seelischen Probleme in den vergangenen 4 Wochen Ihre normalen Kontakte zu Familienangehörigen, Freunden, Nachbarn oder zum Bekanntenkreis beeinträchtigt?

(Bitte kreuzen Sie nur ein Kästchen an)

Überhaupt nicht	Etwas	Mäßig	Ziemlich	Sehr
☐	☐	☐	☐	☐

21. Wie stark waren Ihre Schmerzen in den vergangenen 4 Wochen?

(Bitte kreuzen Sie nur ein Kästchen an)

Ich hatte keine Schmerzen	☐
Sehr leicht	☐
Leicht	☐
Mäßig	☐
Stark	☐
Sehr stark	☐

22. Inwieweit haben die Schmerzen Sie in den vergangenen 4 Wochen bei der Ausübung Ihrer Alltagstätigkeiten zu Hause und im Beruf behindert?

(Bitte kreuzen Sie nur ein Kästchen an)

Überhaupt nicht	☐
Ein bisschen	☐
Mäßig	☐
Ziemlich	☐
Sehr	☐

23. In diesen Fragen geht es darum, wie Sie sich fühlen und wie es Ihnen in den vergangenen 4 Wochen gegangen ist. (Bitte kreuzen Sie in jeder Zeile die Antwort an, die Ihrem Befinden am ehesten entspricht).

Wie oft waren Sie in den vergangenen 4 Wochen

(Bitte kreuzen Sie in jeder Zeile nur ein Kästchen an)

	Immer	Meistens	Ziemlich oft	Manchmal	Selten	Nie
a) … voller Schwung?	☐	☐	☐	☐	☐	☐
b) … sehr nervös?	☐	☐	☐	☐	☐	☐
c) … so niedergeschlagen, dass Sie nichts aufheitern konnte?	☐	☐	☐	☐	☐	☐
d) … ruhig und gelassen?	☐	☐	☐	☐	☐	☐
e) … voller Energie?	☐	☐	☐	☐	☐	☐
f) … entmutigt und traurig?	☐	☐	☐	☐	☐	☐
g) … erschöpft?	☐	☐	☐	☐	☐	☐
h) … glücklich?	☐	☐	☐	☐	☐	☐
i) ... müde?	☐	☐	☐	☐	☐	☐

24. Wie häufig haben Ihre körperliche Gesundheit oder seelischen Probleme in den vergangenen 4 Wochen Ihre Kontakte zu anderen Menschen (Besuche bei Freunden, Verwandten usw.) beeinträchtigt?

(Bitte kreuzen Sie nur ein Kästchen an)

Immer	Meistens	Manchmal	Selten	Nie
☐	☐	☐	☐	☐

25. Inwieweit trifft jede der folgenden Aussagen auf Sie zu?

(Bitte kreuzen Sie in jeder Zeile nur ein Kästchen an)

	Trifft ganz zu	Trifft weitgehend zu	Weiss nicht	Trifft weitgehend nicht zu	Trifft überhaupt nicht zu
a) Ich scheine etwas leichter als andere krank zu werden	☐	☐	☐	☐	☐
b) Ich bin genau so gesund wie alle anderen, die ich kenne	☐	☐	☐	☐	☐
c) Ich erwarte, dass meine Gesundheit nachlässt	☐	☐	☐	☐	☐
d) Ich erfreue mich ausgezeichneter Gesundheit	☐	☐	☐	☐	☐

26. Wie oft haben Sie in der vergangenen Woche Schmerzmittel genommen (auch nicht rezeptpflichtige Mittel)?

(Bitte kreuzen Sie nur ein Kästchen an)

Drei oder mehr als dreimal am Tag	☐
Ein oder zweimal am Tag	☐
Alle paar Tage	☐
Einmal in der Woche	☐
Überhaupt nicht	☐

27. Rauchen Sie?

(Bitte kreuzen Sie nur ein Kästchen an)

Ja	☐
Nein, während der letzten 6 Monate habe ich aufgehört	☐
Nein, ich habe vor länger als 6 Monaten aufgehört	☐
Ich habe nie geraucht	☐

Welche Behandlungsergebnisse erwarten Sie?

(Bitte kreuzen Sie in jeder Zeile nur eine Zahl an)

	Ganz unwahr-scheinlich	Gering wahr-scheinlich	Wahr-scheinlich	Sehr wahr-scheinlich	Höchst wahr-scheinlich	Für mich nicht relevant
**28.** Nachlassen der Beschwerden (Schmerz, Steifheit, Schwellung, Taubheit, Kraftlosigkeit, Instabilität)	1	2	3	4	5	6
**29.** Mehr Alltags-aktivitäten durch-führen zu können	1	2	3	4	5	6
**30.** Besser schlafen zu können	1	2	3	4	5	6
**31.** Zu meinem bisherigen Beruf zurückkehren zu können	1	2	3	4	5	6
**32.** Sport und Freizeit-aktivitäten ausüben zu können	1	2	3	4	5	6
**32a.** Weitere Fähig-keitsstörungen zu vermeiden	1	2	3	4	5	6

33. Wie würden Sie sich fühlen, wenn Sie den Rest Ihres Lebens mit Ihren jetzigen Beschwerden verbringen müssten?

(Bitte kreuzen Sie nur ein Kästchen an)

Sehr unzufrieden ☐

Einigermaßen unzufrieden ☐

Neutral ☐

Einigermaßen zufrieden ☐

Sehr zufrieden ☐

<u>Disabilities of Arm, Shoulder, Hand Modul</u>

<u>ANLEITUNG:</u>

Dieser Fragebogen beschäftigt sich sowohl mit Ihren Beschwerden als auch Ihren Fähigkeiten, bestimmte Tätigkeiten auszuführen.

Bitte beantworten Sie <u>alle Fragen</u> gemäß Ihrem Zustand in der <u>vergangenen Woche</u>, indem Sie einfach die entsprechende Zahl ankreuzen.
Wenn Sie in der vergangenen Woche keine Gelegenheit gehabt haben, eine der unten aufgeführten Tätigkeiten durchzuführen, so wählen Sie die Antwort aus, die Ihrer Meinung nach am ehesten zutreffen würde.
Es ist nicht entscheidend, mit welchem Arm oder welcher Hand Sie diese Tätigkeiten ausüben. Antworten Sie Ihrer Fähigkeit entsprechend, ungeachtet, wie Sie die Aufgaben durchführen konnten.

Bitte schätzen Sie Ihre Fähigkeit ein, wie Sie folgende Tätigkeiten in der <u>vergangenen Woche</u> durchgeführt haben, indem Sie die entsprechende Zahl ankreuzen.

	Keine Schwierig-keiten	Geringe Schwierig-keiten	Mäßige Schwierig-keiten	Erhebliche Schwierig-keiten	Nicht möglich
**34.** Ein neues oder festverschlossenes Glas öffnen	1	2	3	4	5
**35.** Schreiben	1	2	3	4	5
**36.** Einen Schlüssel umdrehen	1	2	3	4	5
**37.** Eine Mahlzeit zubereiten	1	2	3	4	5
**38.** Eine schwere Tür aufstoßen	1	2	3	4	5
**39.** Einen Gegenstand über Kopfhöhe auf ein Regal stellen	1	2	3	4	5
**40.** Schwere Hausarbeit (z. B Wände abwaschen, Boden putzen)	1	2	3	4	5
**41.** Garten- oder Hofarbeit	1	2	3	4	5
**42.** Betten machen	1	2	3	4	5
**43.** Eine Einkaufstasche oder einen Aktenkoffer tragen	1	2	3	4	5

	Keine Schwierig-keiten	Geringe Schwierig-keiten	Mäßige Schwierig-keiten	Erhebliche Schwierig-keiten	Nicht möglich
44. Einen schweren Gegenstand tragen (über 5kg)	1	2	3	4	5
45. Eine Glühbirne über Ihrem Kopf auswechseln	1	2	3	4	5
46. Ihre Haare waschen oder fönen	1	2	3	4	5
47. Ihren Rücken waschen	1	2	3	4	5
48. Einen Pullover anziehen	1	2	3	4	5
49. Ein Messer benutzen, um Lebensmittel zu schneiden	1	2	3	4	5
50. Freizeitaktivitäten, die wenig körper-liche Anstrengung verlangen (z. B. Karten spielen, Stricken, usw.)	1	2	3	4	5
51. Freizeitaktivitäten, bei denen auf Ihren Arm Druck oder ein Stoß ausgeübt wird (z. B. Golf, Hämmern, Tennis, usw.)	1	2	3	4	5
52. Freizeitaktivitäten, bei denen Sie Ihren Arm frei bewegen (z. B. Badminton, Frisbee)	1	2	3	4	5
53. Mit Fortbewegungs-mitteln zurecht zukommen (um von einem Platz zum anderen zu gelangen)	1	2	3	4	5
54. Sexuelle Aktivität	1	2	3	4	5

55. **In welchem Ausmaß haben Ihre Schulter-, Arm- oder Handprobleme Ihren normalen sozialen Aktivitäten mit Familie, Freunden, Nachbarn oder anderen Gruppen während der vergangenen Woche beeinträchtigt?**

Überhaupt nicht	Ein wenig	Mäßig	Ziemlich	Sehr
1	2	3	4	5

56. **Waren Sie in der vergangenen Woche durch Ihre Schulter-, Arm- oder Handprobleme in Ihrer Arbeit oder anderen alltäglichen Aktivitäten eingeschränkt?**

Überhaupt nicht eingeschränkt	Ein wenig eingeschränkt	Mäßig eingeschränkt	Sehr eingeschränkt	Nicht möglich
1	2	3	4	5

Bitte schätzen Sie die Schwere der folgenden Beschwerden während der letzten Woche ein.

(Bitte kreuzen Sie in jeder Zeile die entsprechende Zahl an)

	Keine	Leichte	Mäßige	Starke	Sehr starke
57. Schmerzen in Schulter, Arm oder Hand	1	2	3	4	5
58. Schmerzen in Schulter, Arm oder Hand, während der Ausführung einer bestimmten Tätigkeit	1	2	3	4	5
59. Kribbeln (Nadelstiche) in Schulter, Arm oder Hand	1	2	3	4	5
60. Schwächegefühl in Schulter, Arm oder Hand	1	2	3	4	5
61. Steifheit in Schulter, Arm oder Hand	1	2	3	4	5

62. **Wie groß waren Ihre Schlafstörungen in der letzten Woche, aufgrund von Schmerzen im Schulter-, Arm- oder Handbereich?**

Keine Schwierigkeiten	Geringe Schwierigkeiten	Mäßige Schwierigkeiten	Erhebliche Schwierigkeiten	Nicht möglich
1	2	3	4	5

63. **Aufgrund meiner Probleme im Schulter-, Arm- oder Handbereich empfinde ich meine Fähigkeiten als eingeschränkt, ich habe weniger Selbstvertrauen oder ich fühle, dass ich mich weniger nützlich machen kann.**

Stimme überhaupt nicht zu	Stimme nicht zu	Weder Zustimmung noch Ablehnung	Stimme zu	Stimme sehr zu
1	2	3	4	5

Die folgenden Fragen beziehen sich auf den Einfluss Ihres Schulter-, Arm-, oder Handproblems beim Spielen Ihres Musikinstrumentes oder beim Ausüben Ihres Sports oder beides.

Wenn Sie mehr als ein Instrument spielen oder mehr als eine Sportart ausüben (oder beides), so beantworten Sie bitte die Fragen in bezug auf das Instrument oder die Sportart, die für Sie am wichtigsten ist.
Bitte geben Sie dieses Instrument bzw. Ihre Sportart hier an:

____

Bitte kreuzen Sie die Zahl an, die Ihre körperlichen Fähigkeiten in der vergangenen Woche am besten beschreibt.

Hatten Sie irgendwelche Schwierigkeiten:

	Keine Schwierigkeiten	Geringe Schwierigkeiten	Mäßige Schwierigkeiten	Erhebliche Schwierigkeiten	Nicht möglich
**64.** In der üblichen Art und Weise Ihr Musikinstrument zu spielen oder Sport zu treiben?	1	2	3	4	5
**65.** Aufgrund der Schmerzen in Schulter, Arm oder Hand Ihr Musikinstrument zu spielen oder Sport zu treiben?	1	2	3	4	5
**66.** So gut wie Sie es möchten, Ihr Musikinstrument zu spielen oder Sport zu treiben?	1	2	3	4	5
**67.** Die bisher gewohnte Zeit mit dem Spielen Ihres Musikinstrumentes oder mit Sporttreiben zu verbringen?	1	2	3	4	5

Die folgenden Fragen beziehen sich auf den Einfluss Ihres Schulter-, Arm- oder Handproblems bei Ihrer Arbeit.

Bitte kreuzen Sie die Zahl an, die Ihre körperlichen Fähigkeiten in der vergangenen Woche am besten beschreibt.

Hatten Sie irgendwelche Schwierigkeiten:

	Keine Schwierig-keiten	Geringe Schwierig-keiten	Mäßige Schwierig-keiten	Erhebliche Schwierig-keiten	Nicht möglich
**68.** In der üblichen Art und Weise zu arbeiten?	1	2	3	4	5
**69.** Aufgrund der Schmerzen in Schulter, Arm oder Hand Ihre übliche Arbeit zu erledigen?	1	2	3	4	5
**70.** So gut wie Sie es möchten, zu arbeiten?	1	2	3	4	5
**71.** Die bisher gewohnte Zeit mit Ihrer Arbeit zu verbringen?	1	2	3	4	5

ANGABEN ZUR PERSON

72. Geschlecht:

☐ Männlich

☐ Weiblich

73. Leben Sie:

☐ Allein

☐ Mit Partner / Partnerin

☐ Im Mehrpersonenhaushalt

74. Schulabschluss:

☐ Hauptschule

☐ Mittlere Reife

☐ Abitur

☐ Fachschule

☐ Fachhochschule oder Universität

☐ Abgeschlossene Ausbildung (z.B. Lehre)

☐ Anderes, bitte hier angeben ______________________________

75. Sind Sie:

☐ Verheiratet

☐ Verwitwet

☐ Ledig

☐ Geschieden oder getrennt lebend

76. Wohnen Sie mit jemandem zusammen, der für Sie sorgen kann?

☐ Ja

☐ Nein

BERUFLICHE SITUATION

77. Welche der folgenden Aussagen beschreiben Ihre derzeitige berufliche Situation?

☐ Ich befinde mich zur Zeit in einem Arbeitsverhältnis

☐ Hausfrau oder Hausmann

☐ Im Altersruhestand

☐ Dienstbefreit oder beurlaubt

☐ Arbeitslos

☐ Behindert oder aufgrund einer Erkrankung im Ruhestand

☐ Student/-in

☐ Anderes, bitte angeben ____________________________

78. Beziehen Sie eine der folgenden Unterstützungen oder planen Sie diese zu beantragen?
(mehrfach Angaben sind möglich)

	**Ich beziehe**		**Ich habe beantragt**		**Ich plane zu beantragen**	
**Sozialhilfe**	ja	nein	ja	nein	ja	nein
**Gesetzliche Rente**	ja	nein	ja	nein	ja	nein
**BG-Rente**	ja	nein	ja	nein	ja	nein

79. Abschließend würde uns noch folgendes interessieren:

Welche Probleme beschäftigen Sie zur Zeit am meisten?

1.

2.

3.

Wir bedanken uns für Ihre Mitarbeit!
